# Poly(Ethylene Glycol)-Based Backbones with High Peptide Loading Capacities

**DOI:** 10.3390/molecules191117559

**Published:** 2014-10-30

**Authors:** Aoife O’Connor, Jean-Noel Marsat, Annachiara Mitrugno, Tom Flahive, Niamh Moran, David Brayden, Marc Devocelle

**Affiliations:** 1 Department of Pharmaceutical and Medicinal Chemistry, Centre for Synthesis and Chemical Biology, Royal College of Surgeons in Ireland, 123 St Stephen’s Green, Dublin 2, Ireland; E-Mails: aoifemoconnor87@gmail.com (A.O.); marsatjn@yahoo.fr (J.-N.M.); tomflahive@rcsi.ie (T.F.); 2 Department of Molecular and Cellular Therapeutics, Royal College of Surgeons in Ireland, 123 St Stephen’s Green, Dublin 2, Ireland; E-Mails: annachiaramitrugno@rcsi.ie (A.M.); nmoran@rcsi.ie (N.M.); 3 UCD School of Veterinary Medicine and UCD Conway Institute, University College Dublin, Belfield, Dublin 4, Ireland; E-Mail: david.brayden@ucd.ie

**Keywords:** peptide-polymer conjugates, loading capacity, poly(allyl glycidyl ether), thiol-ene conjugation

## Abstract

Polymer-peptide conjugates are a promising class of compounds, where polymers can be used to overcome some of the limitations associated with peptides intended for therapeutic and/or diagnostic applications. Linear polymers such as poly(ethylene glycol) can be conjugated through terminal moieties and have therefore limited loading capacities. In this research, functionalised linear poly(ethylene glycol)s are utilised for peptide conjugation, to increase their potential loading capacities. These poly(ethylene glycol) derivatives are conjugated to peptide sequences containing representative side-chain functionalised amino acids, using different conjugation chemistries, including copper-catalysed azide-alkyne cycloaddition, amide coupling and thiol-ene reactions. Conjugation of a sequence containing the RGD motif to poly(allyl glycidyl ether) by the thiol-ene reaction, provided a conjugate which could be used in platelet adhesion studies.

## 1. Introduction

Peptides have the potential to be excellent therapeutic and targeting moieties, however their clinical use is limited by their lack of bioavailability, short plasma half-life due to rapid clearance through kidneys, poor stability as a result of enzymatic degradation and potential immunogenicity [1,2]. Effective solutions to improve the delivery and/or pharmacokinetic and pharmacodynamic properties of therapeutic peptides have been sought. Among them, the conjugation to polymers has resulted in multiple applications, not limited to clinical products [3], combining the advantages of these two classes of macromolecules [4]. Several polymer conjugates of low molecular weight drugs [5], peptides [3,4,5,6,7,8] and other macromolecules [9,10,11] based on water-soluble polymers have been developed. 

Polyethylene glycol (PEG) in particular has been successfully exploited to improve the solubility, stability and prevent the toxicity of its cargoes, notably peptides [12,13] and proteins [10]. First and second generation PEGs, based on a linear backbone, are inherently characterised by a low loading capacity. The latter can be increased by the generation of more complex structures, for example, PEG dendrimers and multi-arm PEGs such as star-PEGs. Another option is to polymerise an epoxide monomer yielding a linear polyether backbone reminiscent of PEG, but containing a functional group allowing the conjugation of the cargo along this backbone, or to co-polymerise this monomer with an unfunctionalised ethylene oxide monomer [14]. These homo- and co-polymers should maintain the capacity of the PEG chain to associate with two to three water molecules at each monomeric unit, a property which significantly contributes to the benefits imparted by PEG to biopharmaceutical agents, but also allow the conjugation of multiple copies of this agent through the repeating functional group. Among the functionalised epoxide monomers, allyl glycidyl ether is particularly attractive for the generation of multi-functional PEGs. Indeed, it is not only a protected precursor for the synthesis of linear polyglycerols, which can be modified after polymerisation at the hydroxyl groups along the polyether backbone, but also the starting material to poly(allyl glycidyl ether) (PAGE) [15,16], a polymer which can be directly functionalised through, for example, olefin metathesis or thiol-ene chemistry [16].

The synthesis of PAGE homo-polymers, their functionalisation with a synthetic peptide and their comparison to other PEG-based structures with potential high peptide loading capacities are reported herein.

## 2. Results and Discussion

Short polymers were synthesised through either anionic or cationic polymerisation, by heating an epoxide monomer in the presence of a strong base or cationic initiator, respectively. The length of the polymer was determined by the ratio of initiator used in the polymerisation reaction. It ranged from 10 to 50 repeating units, with an average of 20, for the different polymers synthesised, depending on the monomer(s) used. As the number of peptide copies, in applications exploiting polyvalent interactions of peptide sequences, vary according to the different candidate sequences, no general length of the polymer backbone can be predetermined. The length of the polymers obtained here, allowing the conjugation of up to 20 peptide copies on average was deemed suitable for the application targeted [17]. They were purified by Size-Exclusion Chromatography (SEC) using Sephadex LH20 to remove byproducts and analysed by ^1^H-NMR, MALDI-TOF MS and analytical RP-HPLC where possible.

For the peptide candidates, two sequences containing representative guanidino-, hydroxyl- and carboxyl-, or its amidated counterpart, side-chain functionalised residues were selected for initial conjugation assays. They were a pentapeptide (GRGDS) containing the RGD integrin-recognition motif and a hexapeptide bearing the extracellular amino-terminal domain (SFLLRN, also known as TRAP_1__–6_) of the Protease-activated Receptor-1 (PAR-1), a platelet receptor for thrombin [18]. A third sequence, GWYRGRL, a collagen IIα1 binding peptide, was also chosen for complementary assays, to test the conjugation chemistry in the presence of amino acids with phenolic and indolic side-chains.

### 2.1. Homo- and Co-Polymers of Allyl Glycidyl Ether and Their Functionalisation by the Thiol-Ene Reaction

**Scheme 1:**
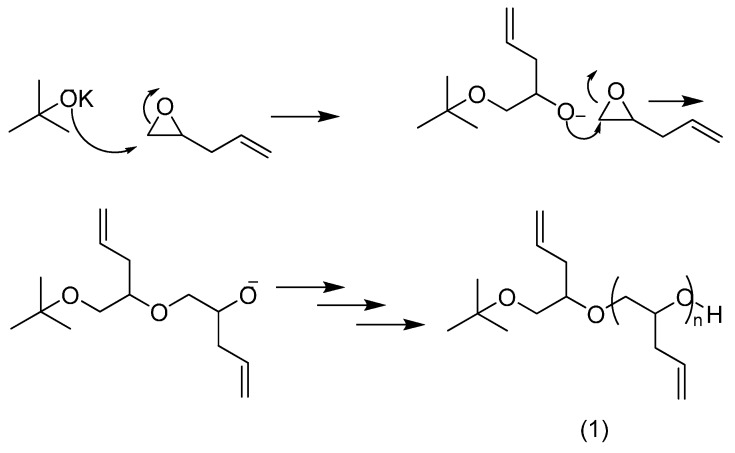
Synthesis of poly(allyl glycidyl ether) (**1**) by anionic polymerisation.

PAGE was synthesized by anionic polymerisation, using potassium *tert*-butoxide as initiator, as shown in Scheme 1.

**Scheme 2:**
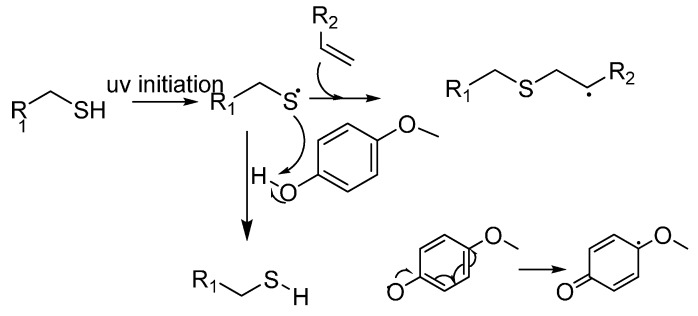
Mechanism of MEHQ quenching of a thiol-radical [19].

Preliminary conjugation assays showed that the selected peptide sequences (RGD and TRAP_1-6_) could be successfully conjugated to this homopolymer by a thiol-ene reaction. The PAGE-TRAP_1-6_ conjugate was poorly soluble in aqueous buffers, therefore, poly(ethylene glycol-*co*-allyl glycidyl ether) co-polymers (P(EO-*co*-AGE)), expected to have greater solubility in water, were prepared by using ethylene carbonate and allyl glycidyl ether as monomers. At that time, P(EO-*co*-AGE)s were independently reported as PEG derivatives with high loading capacities for amino acids and peptides [16]. We were also aware of one limitation of the thiol-ene chemistry encountered during the conjugation of the collagen binding peptide to PAGE, restricting its application to sequences devoid of tyrosine residues and limiting therefore its versatility. The phenolic side-chain of tyrosine can indeed act as a radical scavenger during the thiol-ene reaction, as evidenced by the failure to conjugate GWYRGRL. The reaction was repeated with individual, side-chain functionalised amino acids not present in TRAP_1–6_, namely tyrosine and tryptophan. In the former case, no thiol-ene reaction took place. This is not unexpected, as phenolic groups can be exploited in radical scavengers such as monomethylether hydroquinone (MEHQ), as shown in Scheme 2.

Beside this limitation, the use of a proteinogenic amino acid as one of the partners in the thiol-ene conjugation precludes the presence of free cysteines in the sequence of the bioactive peptide. Additionally, it requires, for heterodetic peptides, the formation of the disulfide bridge(s) and the use of an orthogonally protected cysteine prior to the conjugation step. This could limit the yield of the peptide available for conjugation, when an excess of peptidic reagent is actually required for this reaction.

We therefore investigated alternative conjugation strategies, initially focusing on the copper(I)-catalysed azide-alkyne cyclo-addition, owing to its success in efficient and selective modifications of complex natural macromolecules [20,21,22,23]. Two possible approaches, where the polymer and the peptide were either assigned the role of alkyne or azide partners respectively, were evaluated. We also evaluated an amine-functionalised polymer to provide an alternative route of peptide coupling based on direct amide bond formation.

### 2.2. PEG Derivatives for Multiple Conjugation of Peptides by Azide-Alkyne Cycloaddition

#### 2.2.1. Alkynylated Polymer and Azido-Peptide

**Scheme 3:**
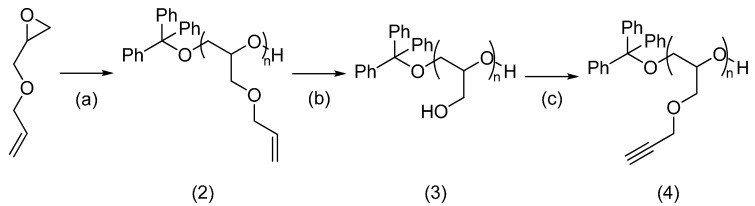
Synthesis of poly(allyl glycidyl ether) (**2**) and conversion to poly(propargyl glycidyl ether) (**4**).

PAGE can also be synthesised by cationic polymerisation and subsequently modified by functional group interconversion. According to this approach, PAGE was first deprotected to give polyglycerol, using palladium over charcoal following previously published methods [24]. Conversion from polyglycerol to poly(propargyl glycidyl ether) was then performed by alkylation with propargyl bromide, as shown in Scheme 3.

Poly(allyl glycidyl ether) (**2**) was obtained in 95% yield from the starting monomer. Full removal of allyl protecting groups was possible, however only 50% of the polymer product was recovered following allyl deprotection. Poly(propargyl glycidyl ether) (**4**) was synthesised with a yield of 15% following purification by SEC through Sephadex LH20.

*Reagents and conditions*: (a) Triphenylcarbenium hexafluorophosphate (0.05 eq.), 0 °C, 12 h; (b) *p*-toluenesulfonic acid (0.1 eq.), 10% Pd/C (40 mg/mmol), reflux, 24 h; (c) propargyl bromide (80% in toluene, 1.8 mmol), NaH, RT, 12 h.

**Figure 1:**
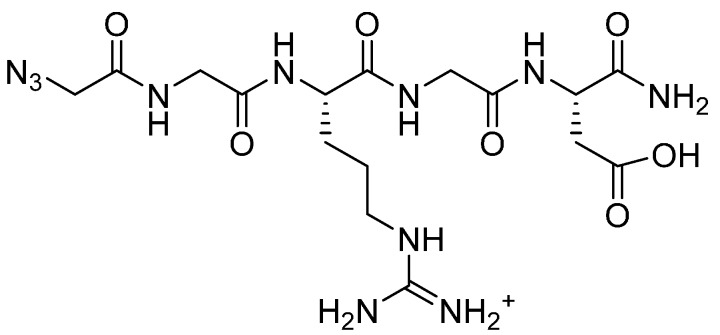
N_3_-GGRGD-NH_2_.

**Figure 2:**
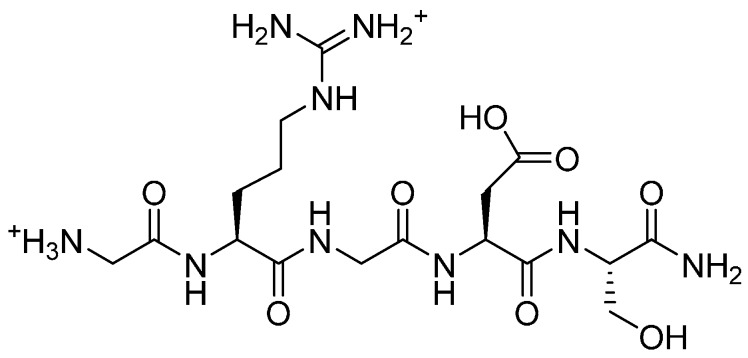
H-GRGDS-NH_2_.

In parallel, two RGD peptide amides were synthesised manually by Solid Phase Peptide Synthesis (SPPS) [16] using the Fmoc/*t*-Bu strategy [25,26], with either an additional *N*-terminal azido glycine, N_3_-GGRGD **5** (Figure 1), or no *N*-terminal modification, GRGDS **6** (Figure 2). Following cleavage from the resin, these peptides were purified by RP-HPLC and analysed by RP- and SEC-HPLC. Compound **5** was intended as the azide partner in the click chemistry reaction with the alkyne polymer **4**. The azido functionality was introduced on the resin-bound peptide by converting its *N-terminus* from amino to azido by reaction with imidazole-1-sulfuryl azide hydrochloride, according to a published procedure applicable to all coded amino acids [27]. Peptide **6** was synthesised as a control peptide sequence for biological testing.

**Scheme 4:**
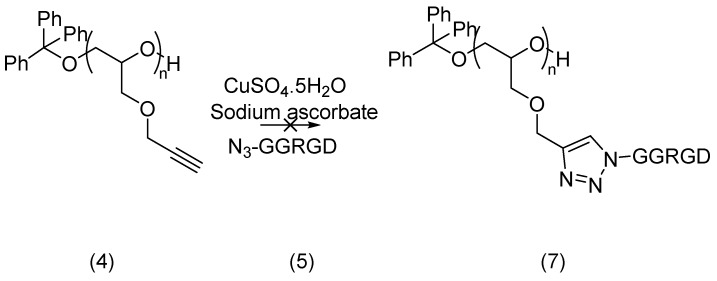
Conjugation attempt of an azido RGD peptide (**5**) with poly(propargyl glycidyl ether) (**4**) in the presence of CuSO_4_·5H_2_O (0.045 eq.), sodium ascorbate (0.045 eq.), water:*t*-butanol (1:1), RT, 48 h.

The cyclo-addition reaction of **4** and **5** was next attempted in the presence of a copper(I) catalyst generated *in situ* from copper sulfate and sodium ascorbate as a reducing agent, and using water and *tert*-butanol as cosolvents (Scheme 4). However, no triazole product, and therefore no conjugation of the peptide on the modified PEG backbone, was obtained under these conditions, as indicated by analysis of the MALDI-TOF MS results. The reaction of polymer **4** with a simpler azide, (2*S*)-2-azido-3-methylbutanoic acid, was also attempted under the same conditions. Following purification by SEC using Sephadex LH-20, the product was analysed by ^13^C NMR, but no signal characteristic of the triazole ring could be observed in its spectrum. This absence of conversion is unexpected as the azide-alkyne cyclo-addition has been successfully used in a number of challenging applications, including polymer chemistry. While the short PEG derivatives produced here could maybe complex the copper, in an analogous manner than crown ethers, this would not explain a complete absence of catalysis, as some conversion is observed when the azide and alkyne partners are inverted (*vide infra*). As no reaction is obtained, even with (2*S*)-2-azido-3-methylbutanoic acid, it is unlikely that this lack of reaction is (solely) due to a reduction of the peptidic azide before conjugation which can occur upon storage of this reagent. This suggests that the reaction deficiency could originate from poly(propargyl glycidyl ether), although the analytical results obtained for this polymer indicated its integrity.

#### 2.2.2. Azido-Polymer and Alkynylated Peptide

**Scheme 5:**
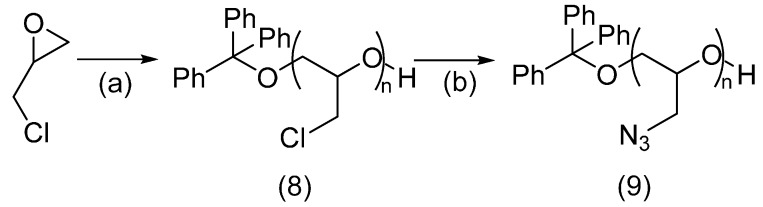
Synthesis of poly(glycidyl azide) (**9**).

Another polymer synthesised for peptide conjugation by azide-alkyne cycloaddition was poly(glycidyl azide) (**9**). Synthesis of polyepichlorohydrin (**8**) by cationic polymerisation, followed by reaction with sodium azide provided this polymeric azide reagent. Quantitative conversion to poly(glycidyl azide) (**9**) took place, with 50% yield of polymer recovered (Scheme 5).

*Reagents and conditions*: (a) Triphenylcarbenium hexafluorophosphate (0.05 eq.), 0 °C, 12 h; (b) sodium azide (1.5 eq.), 100 °C, 12 h.

**Figure 3:**
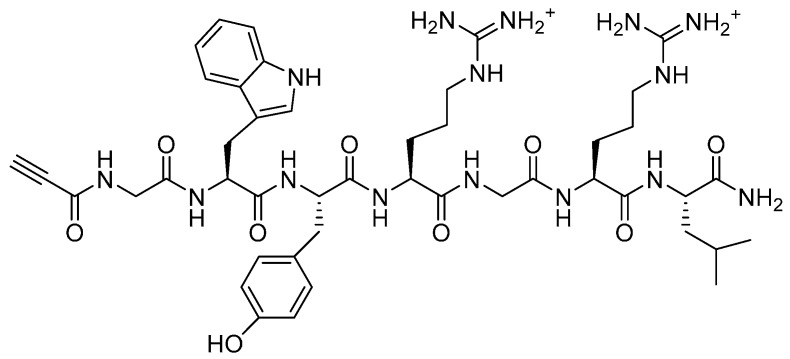
Propiolyl-GWYRGRL-NH_2_.

In parallel, synthesis of an alkynylated collagen-binding peptide was carried out by coupling propiolic acid at the *N-terminus* of the GWYRGRL sequence **10** (Figure 3), using HATU and DIEA [28]. The amide bond formation was monitored by the Kaiser test. The coupling reaction and subsequent cleavage of the alkynylated peptide from the resin were completed in darkness. The later step was performed with a cleavage cocktail consisting of TFA (95% v/v), with triisopropylsilane and water as scavengers (2.5% v/v each), for 90 min, as described for these modified peptides [28]. However, relatively low yields of alkynylated peptide were obtained after purification, the major product being the unmodified peptide. It is possible that the acidity of the terminal alkyne interferes in the coupling reaction performed in the presence of an excess of DIEA. The use of a protected alkyne was therefore investigated to couple the alkyne. However, the reaction performed with trimethylsilane-protected propiolic acid gave similar yields. In addition, complete removal of the TMS group did not occur during cleavage, despite its high susceptibility to water.

**Scheme 6:**
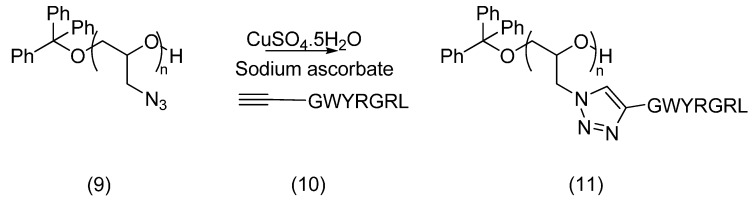
Conjugation of an alkynylated peptide **10** with polyglycidyl azide (**9**) in the presence of CuSO_4_·5H_2_O (0.045 eq.), sodium ascorbate (0.045 eq.), water:*t*-butanol (1:1), RT, 48 h.

Conjugation of the alkyne-modified peptide and polyglycidyl azide was attempted through copper catalysed cycloaddition. Copper sulfate and sodium ascorbate, the most successful reagents documented for this click chemistry, were again selected for the generation of the catalyst. Following conjugation, purification by SEC on Sephadex LH20 was carried out. The reaction shown in Scheme 6 provided a small amount of conjugated product, as evidenced by MALDI-TOF MS analysis which indicated that up to two peptide units had been attached to the polymer backbone.

However, the complete purification of the conjugate obtained remained impracticable, all samples obtained being contaminated by copper traces, as confirmed by the blue/green colour of their solutions.

### 2.3. PEG Derivatives for Multiple Conjugation of Peptides by Amide Linkage

#### Synthesis of Poly(Glycidyl Amine)

**Scheme 7:**
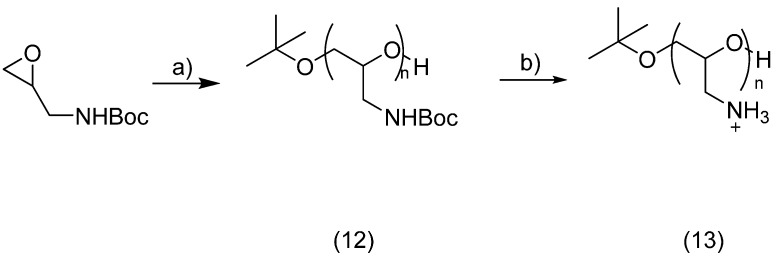
Synthesis of poly(glycidyl amine) (**13**).

Utilising an amide coupling reaction would be the simplest method to conjugate a peptide to the polymer chain, exploiting either a free *N*- or *C*-terminus, despite the limitations imposed on the peptide content related to lysine, or aspartic and glutamic acids, respectively. In this instance, the polymer, conversely, would require carboxylic acid or amine functional groups. Through anionic polymerisation of an amine functionalised epoxide and subsequent deprotection, poly(glycidyl amine) (**13**) was successfully prepared, as shown in Scheme 7.

*Reagents and conditions*: (a) KO*t*Bu, 145 °C, 20 min, (b) TFA: DCM 55:45, RT, 2 h.

**Figure 4:**
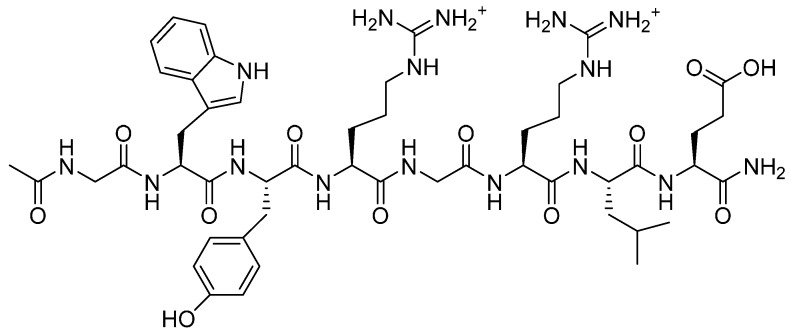
Ac-GWYRGRLE-NH_2_.

**Scheme 8:**
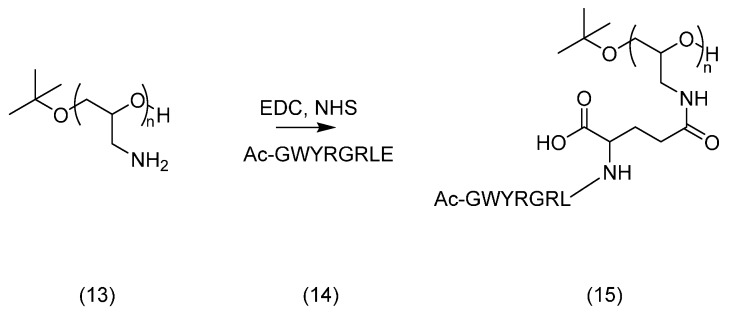
Coupling of poly(glycidyl amine) (**13**) to peptide (**14**).

This was conjugated to a peptide amide with a free carboxylic acid (Ac-GWYRGRLE) **14** (Figure 4). An additional *C*-terminal glutamic acid was added onto the peptide chain to permit conjugation from its γ-carboxyl, as presented in Scheme 8.

Purification by SEC was carried out on Sephadex G-25 and the product was analysed by SEC-HPLC and MALDI-TOF MS. The chromatogram obtained indicated that the coupling reaction had taken place, as evidenced by a new broad signal at lower retention time than the narrow signal corresponding to the peptide alone. From the MALDI-TOF MS, it was determined that a maximum of two peptide chains were attached.

*Reagents and conditions*: EDC (2 mM), NHS (5 mM) at pH 7.5, 12 h.

### 2.4. PEG Derivatives for Multiple Conjugations of Peptides by the Thiol-Ene Reaction

#### 2.4.1. Synthesis

**Scheme 9:**
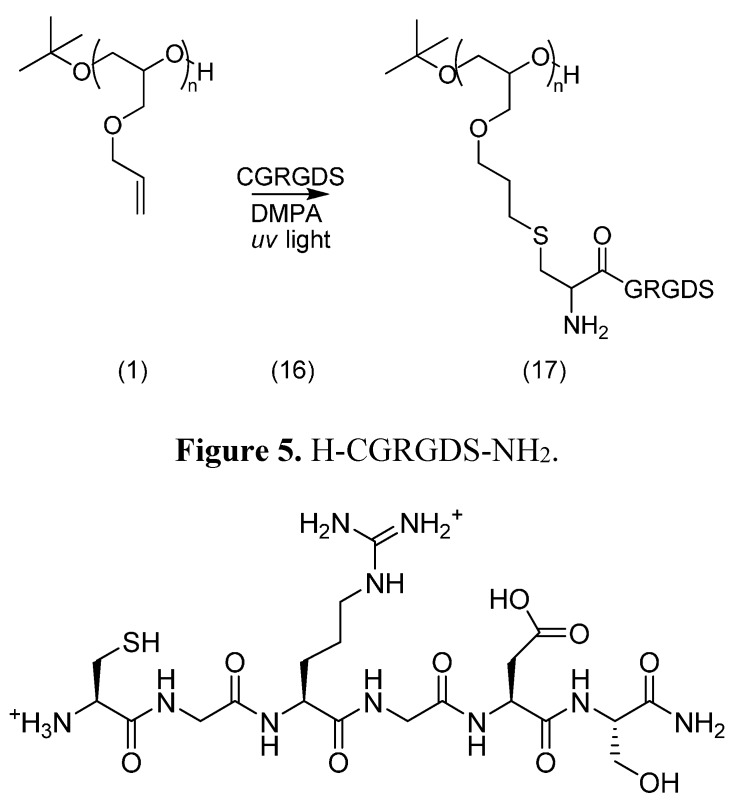
Conjugation of H-CGRGDS-NH_2_ **16** (Figure 5) and poly(allyl glycidyl ether) (**1**) in the presence of DMPA (1 eq.), in DCM under UV light (365 nm), at room temperature, during 48 h.

Having investigated alternative coupling chemistries for the loading of peptides on a PEG backbone, we compared their performances to those achieved with the thiol-ene reaction. The RGD peptide amide was synthesised manually and elongated with a *N*-terminal cysteine. The conjugation reaction was carried out under UV light with dimethyoxyphenylacetophenone (DMPA) as initiator (Scheme 9) and analysed by SEC-HPLC and MALDI-TOF MS. The former technique confirmed the presence of conjugates, larger than the peptide alone and its dimer.

**Figure 5:**
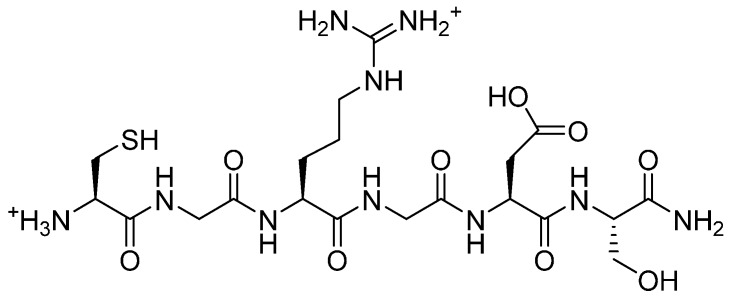
H-CGRGDS-NH_2_.

Following purification by SEC using Sephadex LH-20, MALDI-TOF MS analysis of the product indicated that a polydisperse conjugate containing up to two peptide units had been obtained and that it could be tested in biological assays. This suboptimal multivalency of the peptide ligand, also affecting the previous polymer-peptide conjugates prepared, could originate from the steric hindrance exerted within the functional homopolymer. The formation of peptide homodimers, favoured over the heterodimers formed during conjugation to the polymer, could also impact on the efficiency of the latter reaction. While it can be implemented with an excess of peptide, the number of equivalents of this reagent remains in practice limited by its cost.

#### 2.4.2. Biological Testing

**Figure 6:**
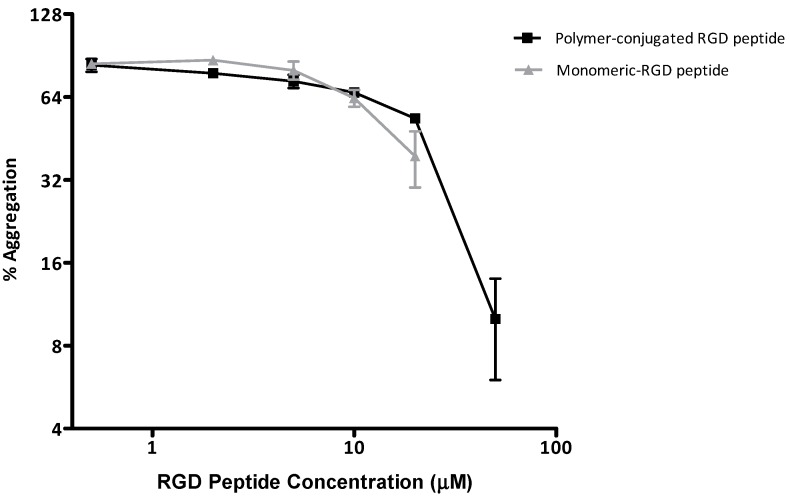
Human washed platelets (300 × 10^3^ platelets/μL) were incubated with increasing concentrations (0.5, 2, 5, 20 and 50 μM) of the free (light trace) or polymeric (dark trace) for 5 min at 37 °C in stirring conditions. Following stimulation with TRAP (4 μM), platelets were assayed for aggregation in light transmission aggregometry. The data are Mean ± SEM of two independent experiments.

Beside its binding to the α_v_β_3_ integrin and therefore its function in the targeting of imaging or therapeutic agents to the tumour vasculature, the RGD (Arg-Gly-Asp) peptide motif is present in plasma fibrinogen and other adhesive proteins and it is recognised by glycoprotein IIb/IIIa (α_IIb_β_3_) on the platelet plasma membrane [29]. Preventing the binding of fibrinogen to α_IIb_β_3_ causes inhibition of platelet aggregation and subsequent thrombus formation. The RGD motif has also been used in research to mimic cell-adhesion proteins [30,31]. These can be on surfaces, to promote adhesion, or may be used in solution, acting as decoys and preventing adhesion in anti-thrombotic therapies [31]. For the studies reported here, various concentrations of peptides were examined for their ability to inhibit platelet aggregation in TRAP-stimulated platelets. The peptides were dissolved in 5% ethanol in water, as they did not fully dissolve in water alone. Both the polymeric and free peptides were effective inhibitors of platelet aggregation. In addition, both acted in a dose-dependent manner as anticipated (Figure 6). 

At a dose of 20 μM the PAGE-RGD conjugate inhibited platelet activation at a lower, albeit not significant, extent when compared with the free peptide. However, when the concentration of the PAGE-RGD conjugate was increased to 50 μM, the platelet aggregation response was completely ablated. Due to solubility problems with the free peptide, it was not possible to test it at 50 μM, thus, Abciximab (Abx, 50 μM), a specific therapeutic chimeric monoclonal blocking antibody against α_IIb_β_3_, was employed as a positive control of the inhibitory effect. The solvent (5% ethanol/water) was also ran as a vehicle control to confirm platelet viability and it did not affect aggregation in this study (data not shown).

## 3. Experimental Section

### 3.1. General Experimental Procedures

Solvents and reagents were purchased from commercial suppliers (Sigma-Aldrich, Wicklow, Ireland; Acros, Dublin, Ireland; Lennox, Dublin, Ireland; and VWR, Dublin, Ireland) and used without further purification unless otherwise stated. ^1^H-NMR spectra were recorded at 400 MHz and ^13^C-NMR spectra at 100.6 MHz on a Bruker Avance 400 spectrometer (Bruker UK Ltd., Coventry, UK). Samples were prepared in CDCl_3_, D_2_O, MeOD and DMSO-d_6_, with these solvent peaks providing internal standards. Chemical shifts (δ_H_) are quoted in parts per million (ppm). Coupling constants (*J*) are in Hertz and corrected to the nearest 0.5 Hz. Multiplicities are denoted by s (singlet), d (doublet), t (triplet), q (quartet), dd (double of doublets), m (multiplets), br (broad), bb (polymer backbone). ^1^H, ^13^C data are included for each compound, where appropriate. MALDI-TOF MS for relevant samples were obtained on an AB Sciex 4800 MALDI-TOF/TOF (AB Sciex UK Ltd., Cheshire, UK), using α-cyano-4-hydroxycinnamic acid (CHCA) as a matrix.

Purification of polymers and conjugates were carried out by SEC using Sephadex LH20 or G25. Sephadex LH20 was run with methanol as a solvent, while Sephadex G25 used water as a solvent. TLC plates were visualised with iodine, ninhydrin or UV fluorescence at the appropriate wavelengths. Analysis of conjugates by SEC was carried out on Varian HPLC Perfusion Chromatography Workstation (Agilent Technologies, Santa Clara, CA, USA) using a Phenomenex BioSep 2000 column. Solvent A was phosphate buffer (0.25 M K_2_HPO_4_ and 0.25 M KH_2_PO_4_) at pH 6 and solvent B was acetonitrile, used at an isocratic elution (50:50) over 45 min.

### 3.2. Polymer Synthesis

*Anionic-polymerisation of poly(allyl glycidyl ether*) (**1**). Potassium *tert*-butoxide (24.5 mg, 0.22 mmol, 1 eq.) and sodium hydride (60% dispersed in mineral oil, 41 mg, 0.22 mmol, 1 eq.) were stirred at room temperature under argon for 1 h. Allyl glycidyl ether (500 mg, 4.39 mmol, 20 eq.) was then added and heated neat at 120 °C for 24 h. Poly(allyl glycidyl ether) (444 mg, 3.89 mmol, 88% yield) was recovered as a viscous yellow-brown gel. ^1^H-NMR (CDCl_3_) δ = 5.85 (m, 1H, OCH_2_CH=CH_2_), 5.28–5.14 (2H, dd, OCH_2_CH=CH_2_), 3.90 (d, 2H), 3.37–3.33 (m, 5H) ^13^C-NMR (CDCl_3_) δ = 134.78 (OCH_2_CH=CH_2_), 117.04 (OCH_2_CH=CH_2_), 79.20 (CH backbone), 72.25 (OCH_2_CH=CH_2_), 70.11 (CH_2_ backbone), 69.54(CH_2_ backbone). MALDI-TOF MS *m*/*z*: repeating unit found 114.0671; requires 114.0681; Δ = 9 ppm.

*Cationic-polymerisation of poly(allyl glycidyl ether)* (**2**). Triphenylcarbenium hexafluorophosphate (85 mg, 0.22 mmol, 0.05 eq.) was dissolved in anhydrous dichloromethane (5 mL) and added dropwise to a solution of allyl glycidyl ether (500 mg, 4.39 mmol, 1 eq.) in dichloromethane (20 mL), cooled to 0 °C. After 12 h stirring, the reaction was quenched with methanol and the solvent evaporated to give poly(allyl glycidyl ether). Poly(allyl glycidyl ether) is a viscous yellow-orange gel, obtained in 95% yield (650 mg, 4.19 mmol). NMR analysis determined the polymer to have an average of 22 units. ^1^H-NMR (CDCl_3_) δ = 7.23 (6H, *m*-Ph), 7.15 (3H, *p*-Ph), 7.05 (6H, *o*-Ph), 5.85 (m, 22H, OCH_2_CH=CH_2_), 5.28–5.14 (44H, dd, OCH_2_CH=CH_2_), 3.90 (d, 44H, OCH_2_CH=CH_2_), 3.63–3.34 (m, 110H, OCH_2_CH(CH_2_R)O) ^13^C-NMR (CDCl_3_) δ = 143. 89 (CR_4_), 134.84 (OCH_2_CH=CH_2_), 130.07 (6C), 128.29 (6C), 126.29 (3C), 117.40 (OCH_2_CH=CH_2_), 79.20 (CH backbone), 72.25 (OCH_2_CH=CH_2_), 70.11 (CH_2_ backbone), 69.54 (CH_2_ backbone); MALDI-TOF MS *m/z*: repeating unit found 114.0671; requires 114.0681; Δ = 9 ppm.

*Polyglycerol* (**3**). To a solution of poly(allyl glycidyl ether) (**1**, 1.68 g, 15 mmol) in methanol (50 mL), *p*-toluenesulfonic acid (285 mg, 1.5 mmol) followed by 10% Pd/C (600 mg, 40 mg per mmol) were added. The solution was refluxed for 24 h. It was then cooled, filtered through a celite plug and rinsed with methanol. The sample was concentrated, redissolved in minimum volume of methanol and the polymer was precipitated with cold diethyl ether. 1.145 g of polymer was recovered as a viscous yellow gel (50% yield). ^1^H-NMR (CDCl_3_) δ = 3.65–3.56 (m, 3H), 3.54–3.47 (m, 2H) ^13^C-NMR (CDCl_3_) δ = 79.69 (br, CH_2_), 68.86 (br, CH), 60.62 (br, CH_2_); MALDI-TOF MS *m/z*: repeating unit found 74.0282 average; requires 74.0368; Δ = 115 ppm.

*Poly(propargyl glycidyl ether)* (**4**). Polyglycerol (**3**, 74 mg, 1 mmol) was dissolved in DMF (1.5 mL) under nitrogen. Sodium hydride, 60% solution dispersed in mineral oil (40 mg, 1 mmol) was added and the solution stirred at room temperature for 30 min. Propargyl bromide, 80% solution in toluene (148 mg, 111 μL, 1 mmol) was added and the solution stirred at room temperature overnight. Water (40 mL) was added, the resulting solution acidified and product was extracted with ether (3 × 15 mL). 21 mg (18% yield) of a thick yellow gel was recovered. ^1^H-NMR (CDCl_3_) δ = 4.14 (s, 2H, OCH_2_CCH), 3.65–3.45 (m, 5H, OCH_2_CH(CH_2_OR)O), 2.40 (1H, OCH_2_CCH) ^13^C-NMR (CDCl_3_) δ = 79.88 (CH_2_CCH), 78.58 (CH_2_CCH), 76.59 (CH_2_OCH_2_CCH), 74.73 (CH(CH_2_OR)), 69.74 (CH_2_CH(CH_2_OR)), 58.59 (CH_2_CCH); MALDI-TOF MS *m/z*: repeating unit found 112.0497; requires 112.0524; Δ = 24 ppm.

*Poly(epichlorohydrin)* (**8**). Epichlorohydrin (1.24 g, 13.5 mmol) was cooled to 0°C. Triphenylcarbenium hexafluorophosphate (133 mg, 0.343 mmol) was dissolved in anhydrous DCM (4 mL) and cooled. This solution was added to the cooled epichlorohydrin over 5 min. It was stirred at room temperature overnight and precipitated with methanol. After centrifuging the solution, a clear gel is formed. This is dried under vacuum to yield poly(epichlorohydrin) as a clear gel (744 mg, 62% yield). NMR analysis determines the polymer to have an average length of 38 units. ^1^H-NMR (CDCl_3_) δ = 7.26 (m, Ph_3_), 3.62 (m, 39H), 3.64–3.53 (m, 26H) ^13^C-NMR (CDCl_3_) δ = 126.92 (Ph_3_), 78.08(br, CH_2_CHO), 69.45 (br, CH_2_CHO), 42.52 (br, CH_2_Cl); MALDI-TOF MS *m/z*: rRepeating unit found: 92.005; requires 92.003; Δ = 22 ppm.

*Poly(glycidyl azide)* (**9**). Poly(epichlorohydrin) (250 mg, 2.72 mmol) was dissolved in DMF (1 mL). Sodium azide (211 mg, 3.25 mmol) was added and the solution was stirred at 100 °C for 12 h. Water (1 mL) was added dropwise after cooling to room temperature and the polymer precipitated out. The gel obtained after centrifuging was dried under vacuum to yield a viscous yellow gel (134 mg, 50% yield). NMR analysis determines the polymer to have an average length of 46 units. ^1^H-NMR (CDCl_3_) δ = 7.22 (m, Ph_3_), 3.30 (m, 150H), 3.25 (m, 93H) ^13^C-NMR (CDCl_3_) δ = 127.92 (Ph_3_), 78.81 (br, CH_2_CHO), 70.77 (br, CH_2_CHO), 52.30 (br, CH_2_N_3_); MALDI-TOF MS *m/z*: repeating unit found 99.042; requires 99.043; Δ = 10 ppm.

*Poly(glycidyl tert-butyl carbamate)* (**12**). To oxiranylmethyl-carbamic acid-*tert*-butyl ester (430 mg, 2.49 mmol), potassium *tert*-butoxide (14 mg, 0.124 mmol) was added neat under nitrogen and the solution was stirred at 145 °C for 20 min. When the polymer had begun to solidify the polymerisation was quenched by adding methanol (2 mL). The solvent was removed under vacuum and 406 mg of product was obtained (94% yield) as a viscous brown gel. NMR analysis, based on the *tert*-butyl group signal determines the polymer to have an average length of 12 units. ^1^H-NMR (CDCl_3_) δ = 3.68–3.23 (m, 60H), 1.37 (s, 108H, 3CH_3_) 1.11 (s, 9H, 3CH_3_ *t*-Bu) ^13^C-NMR (CDCl_3_) δ = 158.5 (br, C=O), 78.6 (br, CH), 69.3 (br, CH_2_), 40.6 (br, CH_2_), 28.34 (3CH_3_), 27.56 (*t*-Bu initiator); MALDI-TOF MS *m/z*: Not obtained as polymer was not readily ionized.

*Poly(glycidyl amine)* (**13**). Amine deprotection was carried out by dissolving the polymer (400 mg, 2.3 mmol) in dichloromethane (2 mL) and trifluoroacetic acid (2 mL). This was stirred at 0 °C for 30 min followed by heating to room temperature for 2 h with no stopper on the flask. The solvent was then evaporated under nitrogen gas, the residue dissolved in water and freeze dried to remove residual acid. A viscous yellow gel was recovered in 100% yield. ^1^H-NMR (CDCl_3_) δ = 3.74–3.53 (br, 36H), 3.33–3.05 (br, 24H), 1.11 (s, 9H, 3CH_3_ *t*-Bu) ^13^C-NMR (CDCl_3_) δ = 75.14 (m, CH), 67.84 (m, CH_2_), 41.49 (m, CH_2_); MALDI-TOF MS *m/z*: repeating unit found average 73.0483; requires 73.0528; Δ = 61 ppm.

### 3.3. General Procedure for Peptide Syntheses

Peptide sequences were assembled manually by SPPS using a fritted syringe. 1 mmol (10 fold molar excess) quantities of each protected amino acid were used. The amine group on the resin is Fmoc protected initially but is available for amino acid coupling at the start of the synthesis, as a result of piperidine (20% in NMP) deprotection. The peptide was elongated from the C-terminus (amide functional group) to the *N-terminus * (amino group).

Following assembly and modification of the sequence, when relevant, the peptide was cleaved from the resin and deprotected of its semi-permanent groups. Generally the following mixture was added to the dried resin with a magnetic stirrer: Trifluoroacetic acid (95%), triisopropylsilane (2.5%), water (2.5%), the reaction was stirred gently for 2.5 h. The solution was then filtered and the peptide precipitated from the filtrate with chilled diethyl ether, followed by isolation of the precipitate by centrifugation (5 min × 2.8 × 10^3^ rpm). The liquid phase was discarded and the peptide washed with ether twice more. The peptide was finally solubilised in water and lyophilised on an EC Modulyo-230 freeze dryer (Fischer Scientific Ireland, Dublin, Ireland).

Peptides were purified by Reverse-Phase HPLC on a PerSeptive Biosystems BioCAD SPRINT (Life Technologies, Warrington, UK) system using a semi preparative column Gemini C-18 reverse-phase chromatography obtained from Phenomenex (Phenomenex, Macclesfield, UK). The Gemini C 18 (250 mm × 10 mm) column was used at 5 mL/min on a linear gradient program from 5% to 65% solvent B. Solvent A was water (0.1% TFA) and solvent B was acetonitrile (0.1% TFA). UV monitoring was performed at 214 nm unless otherwise stated. Analysis of peptides was carried out by Reverse-Phase HPLC on a Varian HPLC Chromatography Workstation (Agilent Technologies, Santa Clara, CA, USA) using the same solvent system as above, at 1 mL/min.

*N_3-_GGRGD-NH_2_* (**5**). Following assembly of peptide sequence GGRGD by SPPS as described above, the terminal amine was converted to N_3_ with imidazole-1-sulfonyl azide hydrochloride (3 eq.), DIEA (4.5 eq.) in DMF. After stirring for 90 min, a Kaiser test determined incomplete coupling and the reaction was repeated with fresh reagents. MALDI-TOF MS *m/z*: found 486.148; requires 485.206. A purity of 82% was determined by RP-HPLC (C18).

*H-GRGDS-NH_2_* (**6**). Prepared as described above. MALDI-TOF MS *m/z*: found 490.48; requires 489.47. RP-HPLC (C18) shows purity of 93%.

*Propiolyl-GWYRGRL-NH_2_* (**10**). Following assembly of the peptide sequence GWYRGRL by SPPS as described above, propiolic acid (5 eq.), HATU (4.9 eq.) and DIEA (12 eq.) were added to the deprotected peptide on resin. As the propiolic is light sensitive, the coupling reaction was completed in the dark for 120 min. The peptide was then cleaved and purified as described above. MALDI-TOF and ESI-MS both confirmed the successful synthesis of this peptide. ESI MS *m/z*: found [M+H]^+1^ 958.46; requires 957.50. A purity of 97% was determined by RP-HPLC (C18).

*Ac-GWYRGRLE-NH_2_* (**14**). Prepared as described above. MALDI-TOF MS *m/z*: found 1077.54; requires 1077.21.RP-HPLC (C18) shows purity of this peptide to be 98%.

*H-CGRGDS-NH_2_* (**16**). Prepared as described above. MALDI-TOF MS: *m/z*: found 593.199; requires 592.63. RP-HPLC (C18) shows purity of 70%.

### 3.4. Conjugations

#### 3.4.1. Cycloaddition Product **7**

Poly(propargyl glycidyl ether) (**4**, 6.1 mg, 1 eq.) was dissolved in *t*-butanol (0.5 mL) and N_3_-GGRGD-NH_2_ (**5**, 53 mg, 2 eq.) was added in water (0.3 mL). CuSO_4_.5H_2_O (6.1 mg, 0.045 eq.) and sodium ascorbate (4.85 mg, 0.45 eq.) were dissolved in water (1 mL each) and 100 μL of each was added to the solution. The resulting mixture was stirred for 48 h at room temperature. MALDI-TOF MS did not give the correct mass for the conjugated product. This conjugation was not considered to be successful.

#### 3.4.2. Cycloaddition Product **11**

Poly(glycidyl azide) (**9**, 3.76 mg, 2 eq.) and propiolyl-GWYRYGL-NH_2_ (**10**, 18 mg, 1 eq.) were dissolved in *tert*-butanol (500 μL) and water (300 μL). Sodium ascorbate (198 mg in 1 mL) and CuSO_4_·5H_2_O (45 mg in 1 mL) solutions were prepared fresh and 100 μL of each was added to the polymer-peptide solution. This was stirred at 30 °C overnight over which period the suspension cleared. The solvent was evaporated and NMR and size exclusion chromatography carried out. MALDI-TOF MS analysis indicated that up to two units of peptide were attached.

#### 3.4.3. Amide Linkage Conjugated Product **15**

Ac-GWYRGRLE-NH_2_ (**14**, 11 mg, 0.01 mmol) was dissolved in 2-(*N*-morpholino)ethanesulfonic acid buffer (0.5 mL) and NHS (10 mg) and EDC (16 mg) were added. The solution was stirred at room temperature for 30 min. Poly(glycidyl amine) (**13**, 0.6 mg, 0.008 mmol) was dissolved in PBS buffer (0.5 mL). It was added to the solution which was stirred overnight. The mixture was purified by Sephadex G-25 and the fractions containing some product as determined by iodine staining were freeze dried. SEC-HPLC and MALDI-TOF MS analyses indicated that the peptide had coupled to the polymer, up to two peptide units per chain. Approximately 1 mg (0.0004 mmol) was recovered, 5% yield.

#### 3.4.4. Thiol-Ene Conjugation Product **17**

To a solution of poly(allyl glycidyl ether) (**1**, 308 mg, 2.7 mmol) in dry DCM (2 mL) in a clear vial, H-CGRGDS-NH_2_ (**16**, 2 eq.) was added in dry methanol (1 mL). 2,2-Dimethoxy-acetophenone (300 mg) was added and stirred under UV light at 365 nm for 48 h. The solvents were evaporated and the conjugate was purified on a Sephadex LH-20 column, eluted with methanol. The conjugate was analysed by NMR, MALDI-TOF MS and SEC-HPLC (Biosep S2000, Phenomenex, Cheshire, UK, 300 mm × 7.8 mm, 5 μm).

### 3.5. Biological Assays

#### 3.5.1. Platelet Isolation

Washed platelets (WP) were prepared from donors who gave informed consent and declared that they were medication free for the previous 10 days. Venous blood was drawn into 15% (v/v) of acidcitrate-dextrose (ACD) anticoagulant (38 mM citric acid anhydrous, 75 mM sodium citrate, 124 mM dextrose). Blood was centrifuged at 150× *g* for 10 min at room temperature and the supernatant platelet rich plasma (PRP) was collected. PRP was acidified with ACD at pH 6.5 and 1 mM prostaglandin E1 (PGE1) was added. The platelets were pelleted 720× *g* for 10 min and resuspended in buffer (6 mM dextrose, 130 mM NaCl, 9 mM NaHCO_3_, 10 mM sodium citrate, 10 mM Tris base, 3 mM KCl, 0.81 mM KH_2_PO_4_ and 0.9 mM MgCl_2_·6H_2_O, pH 7.35) and the concentration adjusted to 3 × 105 platelets/mL. Washed platelets were supplemented with 1.8 mM CaCl_2_.

#### 3.5.2. Platelets Adhesion Assay

Plates (96 transparent flat bottom wells) were coated with fibrinogen (10 mg/mL) 100 μg/mL in phosphate buffered saline (PBS), for 2 h at 37 °C. Nonreactive sites were blocked with bovine serum albumin (BSA 2 mg/mL, 5% in PBS) (1 h and 30 min, 37 °C). Wells were washed twice with 100 μL of JNL Buffer and exposed to platelets. Control surfaces were coated with albumin BSA. Platelets (50 μL, 3 × 105 platelets/mL) were pre-treated with polymer-conjugated RGD peptide (50 μM, 20 μM), monomer RGD peptide (20 μM), Abciximab (Abx, 50 μg/mL) or vehicle control before seeding in each respective well and allowed to adhere for 40 min at 37 °C. Non-adherent platelets were removed, and the wells were rinsed three times with 200 μL of JNL Buffer. The remaining cells were allowed to react with p-nitro phenol phosphate (PnPP) solution (0.1 M sodium acetate, 0.1% Triton X-100) for 90 min at 37 °C. The reaction was quenched by adding 20 μL of sodium hydroxide (NaOH, 1 M) to each well. Adherent platelets were quantified on a Wallac reader at 405 nm. Experiments were repeated at least three times.

#### 3.5.3. Platelet Aggregation Assay

Platelet aggregations were performed at 37 °C in a BioData PAP-4 aggregometer (Horsham, PA, USA). Briefly, 200 µL of washed platelets (250 × 103/µL) were stirred at 37 °C for 2 min, followed by addition of TRAP (SFLLRN; 4 μM) for a further 20 min. To study the effect of peptides, platelets were pre-incubated for 5 min with polymer-conjugate RGD peptide (50 μM, 20 μM) or monomeric RGD peptide (20 μM). Abx (2 mg/mL) was used as positive control and water 5% ethanol as vehicle control. Experiments were repeated at least twice.

## 4. Conclusions

The platelet aggregation study performed with a PEG derivative modified with a synthetic peptide suggests that both the free peptide and its polymeric counterpart can inhibit platelet adhesion. While the efficiency of the polymeric-RGD doesn’t exceed in these studies the efficiency of the monomeric peptide, it is important to remember that in a biological system it is possible that this unsurpassed efficiency may still be offset by the increased stability and circulation time of a polymer conjugate [32]. Therefore, further studies to demonstrate the applicability of the polymeric RGD peptide should examine for example its proteolytic stability in plasma and blood.

Another important factor in these studies is the solubility of these peptides. Both free and polymeric-RGD suffer from low water solubility and were dissolved in 5% ethanol in water, but the peptide still benefits from the conjugation to the polymer backbone. Altering the peptide sequence to include more hydrophilic residues may be of benefit, as would the deprotection of unconjugated allyl groups on the polymer chain, to give water-soluble polyglycerol units along the backbone.

PEG derivatives with high peptide loading capacities are not only potential nanomedicines, but can also find applications as targeted drug delivery systems, by using homing peptides, or polymeric prodrugs, by using protease-dependent peptide linkers. The conjugation chemistry and its efficiency will determine the synthetic strategy for the generation of these candidates and its applicability. The results of the conjugation assays reported here show that the thiol-ene reaction and azide-alkyne cyclo-addition can be implemented, but that some limitations may apply. In both cases, the number of peptide chains appended to the polymer chain is low, approximately 1 peptide for every 10 repeating units, but this could be due to the steric hindrance exerted between adjoining functional groups for conjugation in a homopolymer. This limitation could be addressed by the use of co-polymers such as P(EO-*co*-AGE) or their derivatives obtained by deprotection of the allyl groups and functional group interconversion, as reported here. Both homo- and co-polymers obtained by these approaches have an average molar mass significantly lower than conventional linear PEGs, but it is possible to use one such PEG as an initiator of anionic polymerisation of allyl glycidyl ether, as successfully performed in our studies with a MeO-PEG-OH of average molar mass of 1000 g/mol.

Conjugation chemistries capable of selectively and efficiently loading multiple copies of a peptide to an appropriately functionalised polymeric backbone are not limited to the ones reported here. For example, polyglycerol obtained by allyl deprotection of PAGE can potentially be used for imine-type conjugation chemistries, by oxidation of the polymer’s hydroxyl groups and the use of an oxime- or hydrazide- modified peptide. However, preliminary assays completed during our studies indicated that the polyglycerol remained refractory to oxidation under common conditions, including reagents such as potassium permanganate.

In conclusion, the thiol-ene reaction, despite a restriction in the peptide content limited to tyrosine, is a promising chemistry for the conjugation of peptides to polymeric and other macromolecular materials, owing to the simple scale-up of the peptide synthesis phase, the ease of execution of the conjugation step and the uncomplicated purification of the conjugates produced.

## Supplementary Materials

Supplementary materials can be accessed at: http://www.mdpi.com/1420-3049/19/11/17559/s1.

## Supplementary Files

Supplementary File 1
