# Supplementary material for: Poly(Ethylene Glycol)-Based Backbones with High Peptide Loading Capacities
Source: Molecules. 2014 Oct 30;19(11):17559–77. doi: 10.3390/molecules191117559 (PMC6270934; doi:10.3390/molecules191117559)
Supplement: Supplementary File 1 [file molecules-19-17559-s001.pdf]

Supplementary Materials

Figure S1. Poly(allyl glycidyl ether) (**1**): (**a**) ^{1}H-NMR; (**b**) ^{13}C-NMR; (**c**) MALDI-TOF MS.

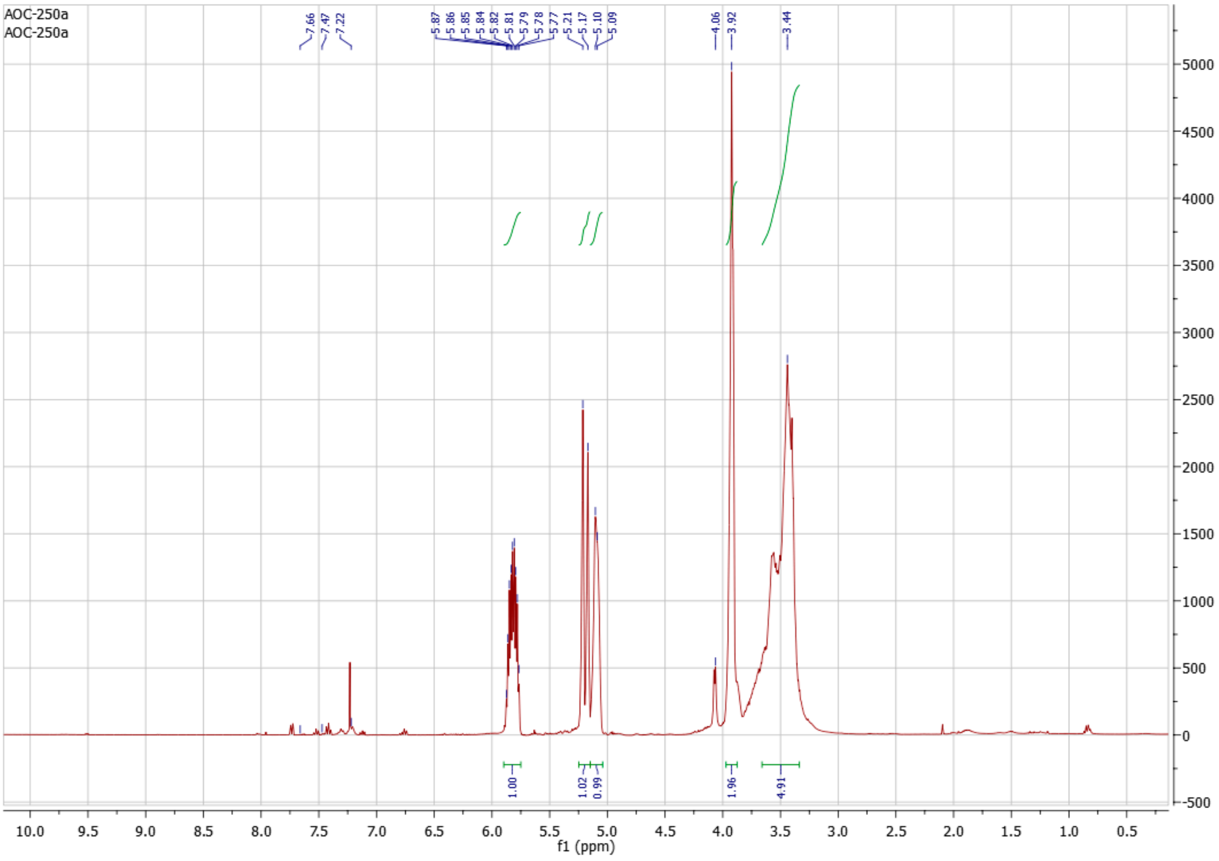

(**a**)

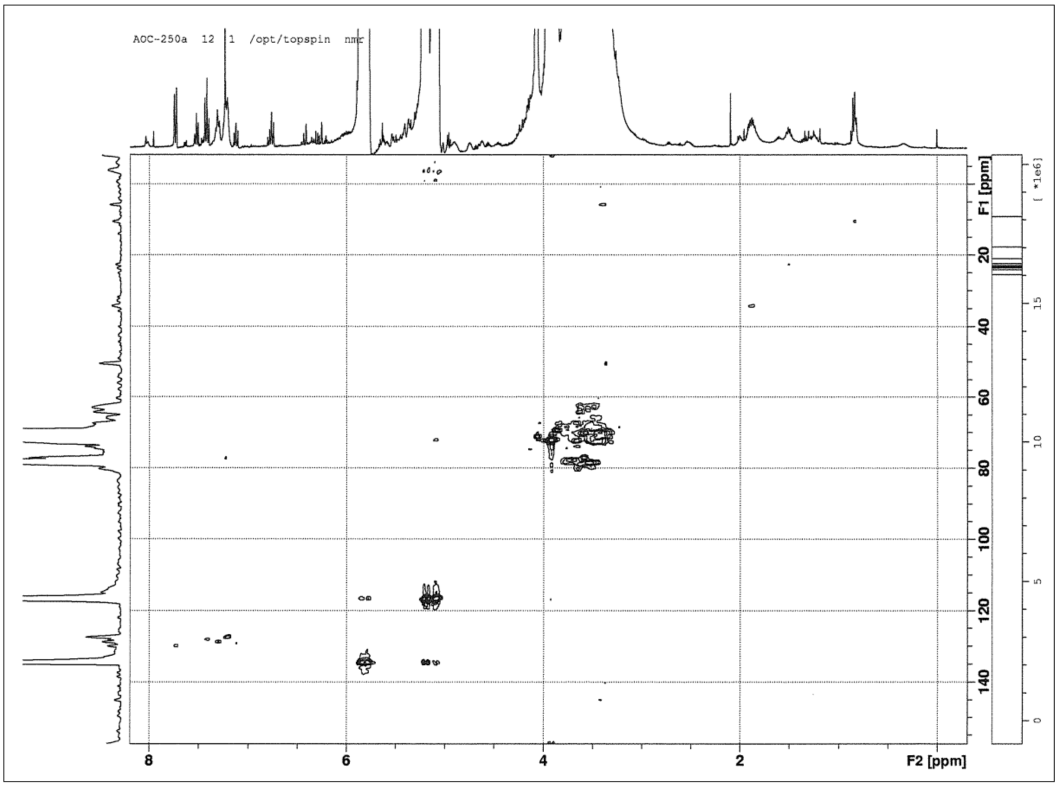

(**b**)

Figure S1. *Cont.*

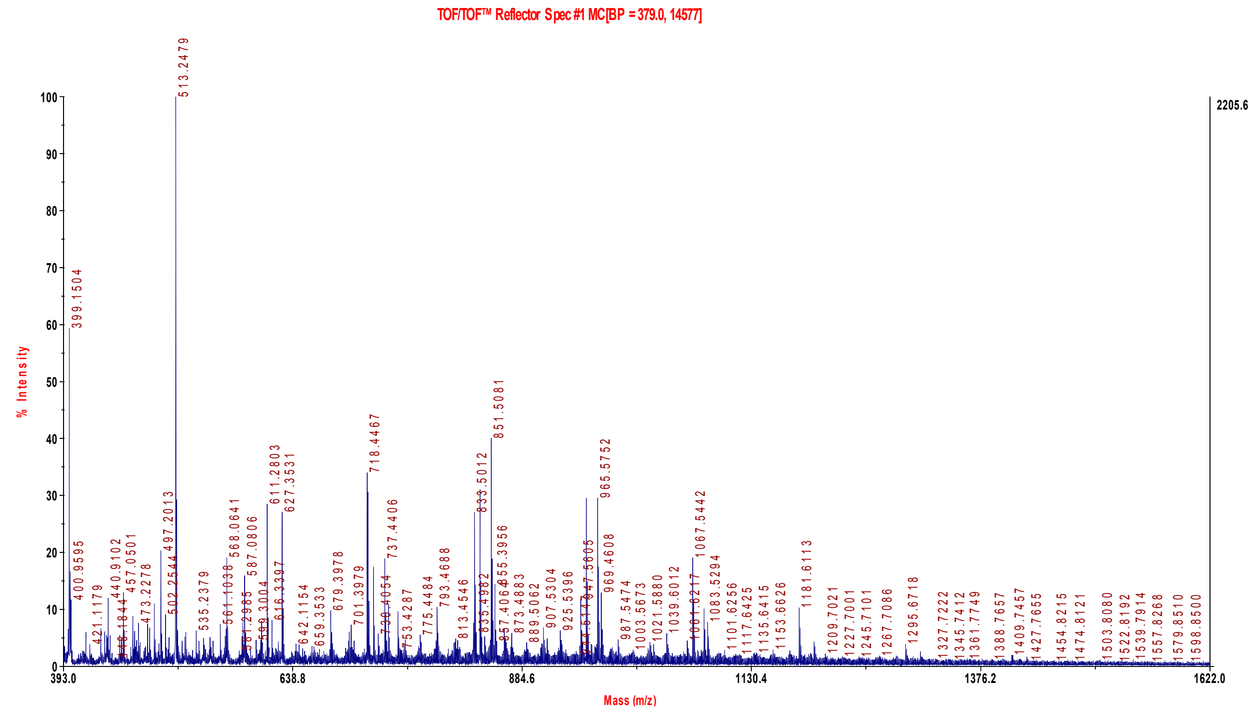

(**c**)

Figure S2. Cationic poly(allyl glycidyl ether) (**2**): (**a**) ^{1}H-NMR; (**b**) ^{13}C-NMR.

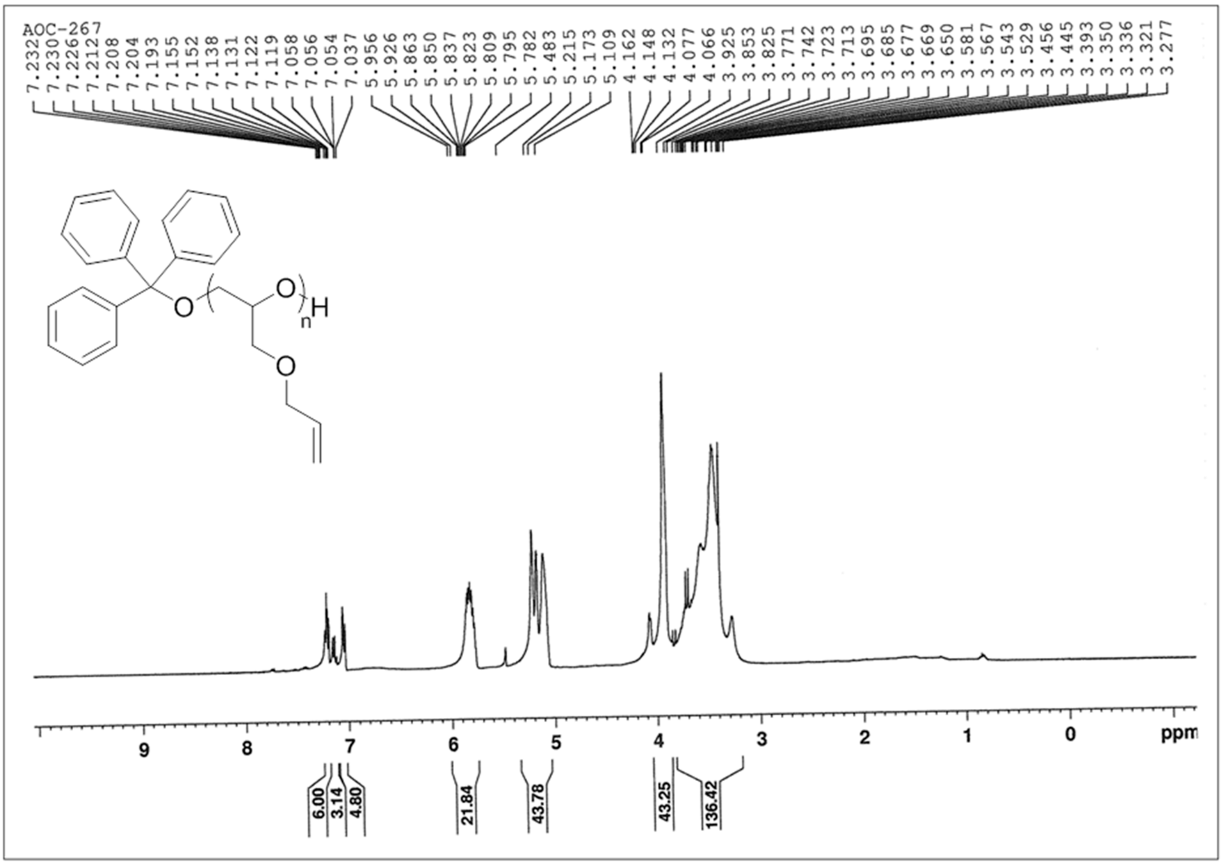

(**a**)

Figure S2. *Cont.*

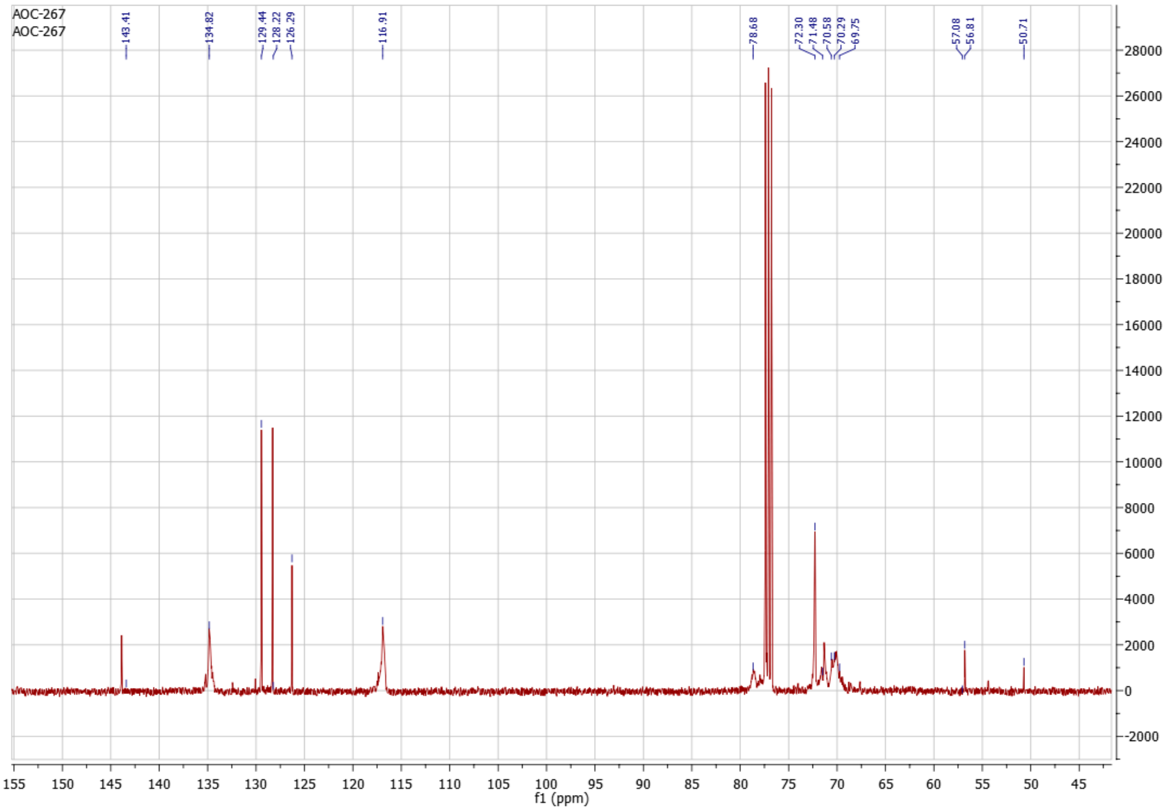

(**b**)

Figure S3. Polyglycerol (**3**): (**a**) ^{1}H-NMR; (**b**) ^{13}C-NMR; (**c**) MALDI-TOF MS.

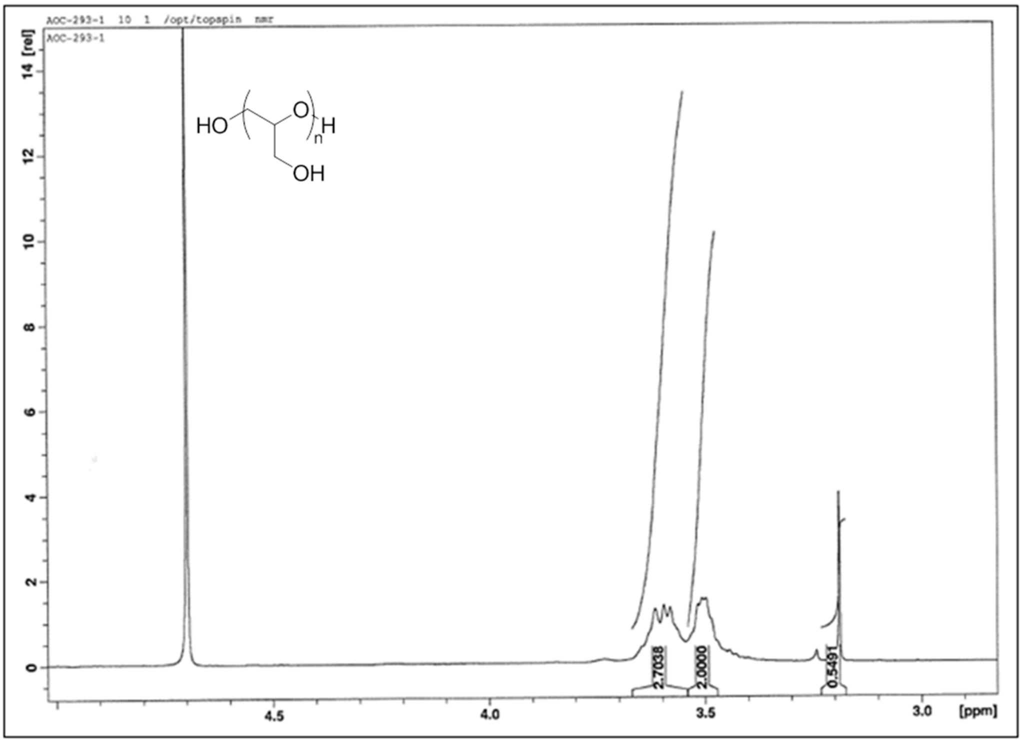

(**a**)

Figure S3. *Cont.*

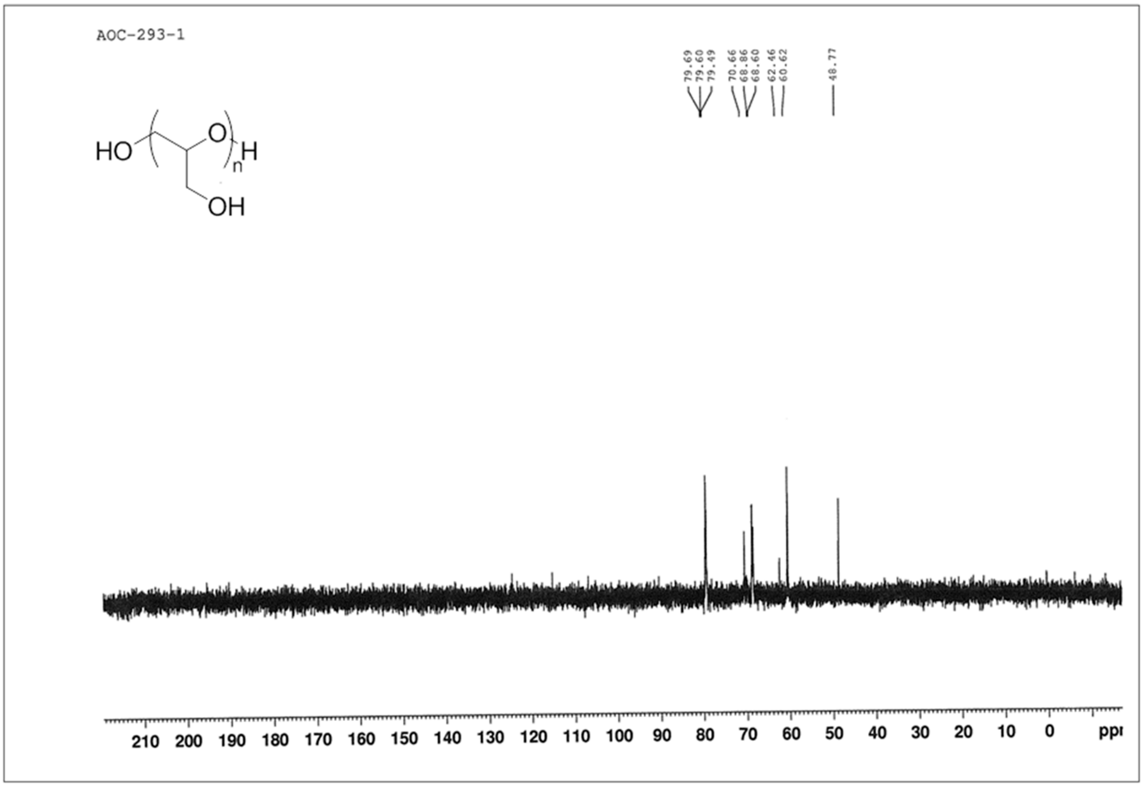

(**b**)

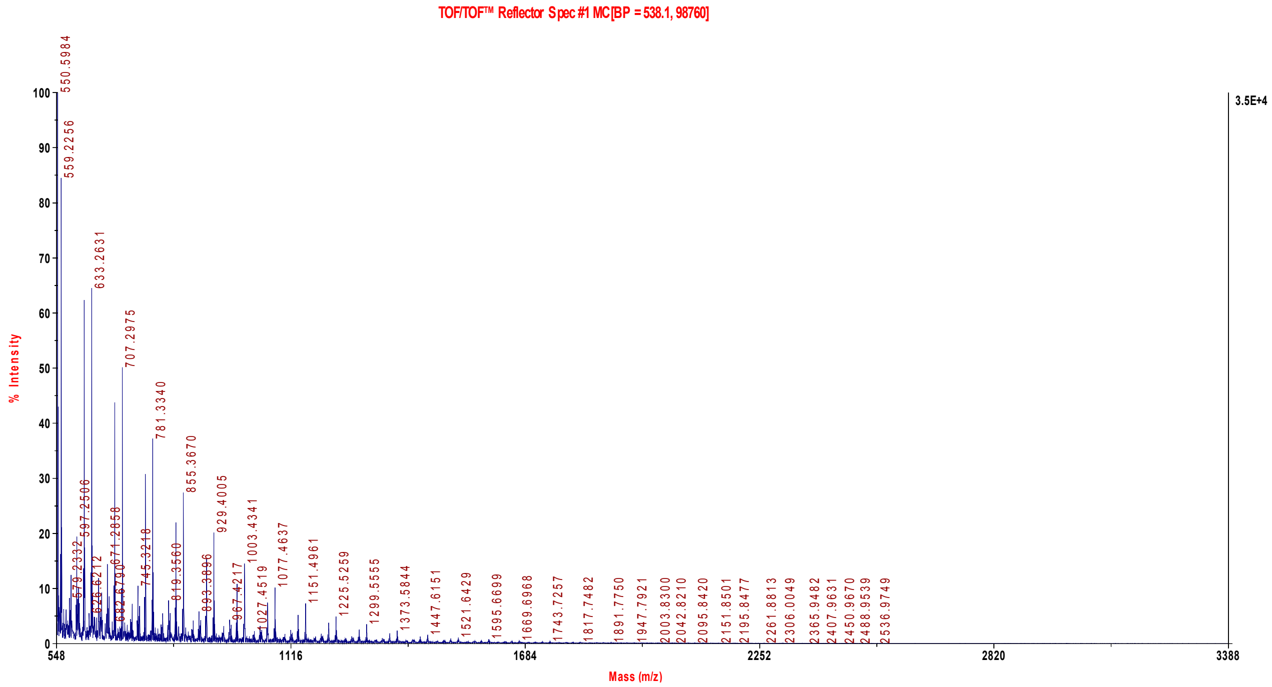

(**c**)

Figure S4. Poly(propargyl glycidyl ether) (**4**): (**a**) ^{1}H-NMR; (**b**) ^{13}C-NMR; (**c**) MALDI-TOF MS.

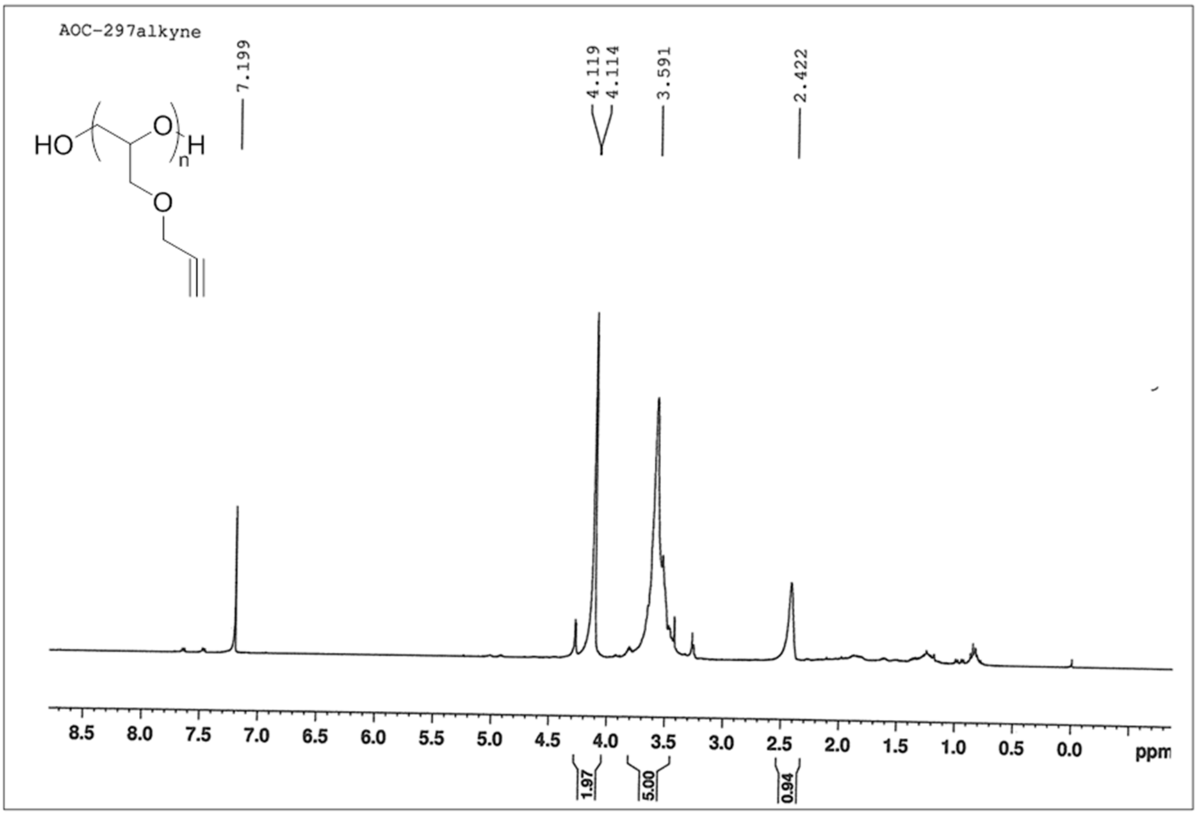

(**a**)

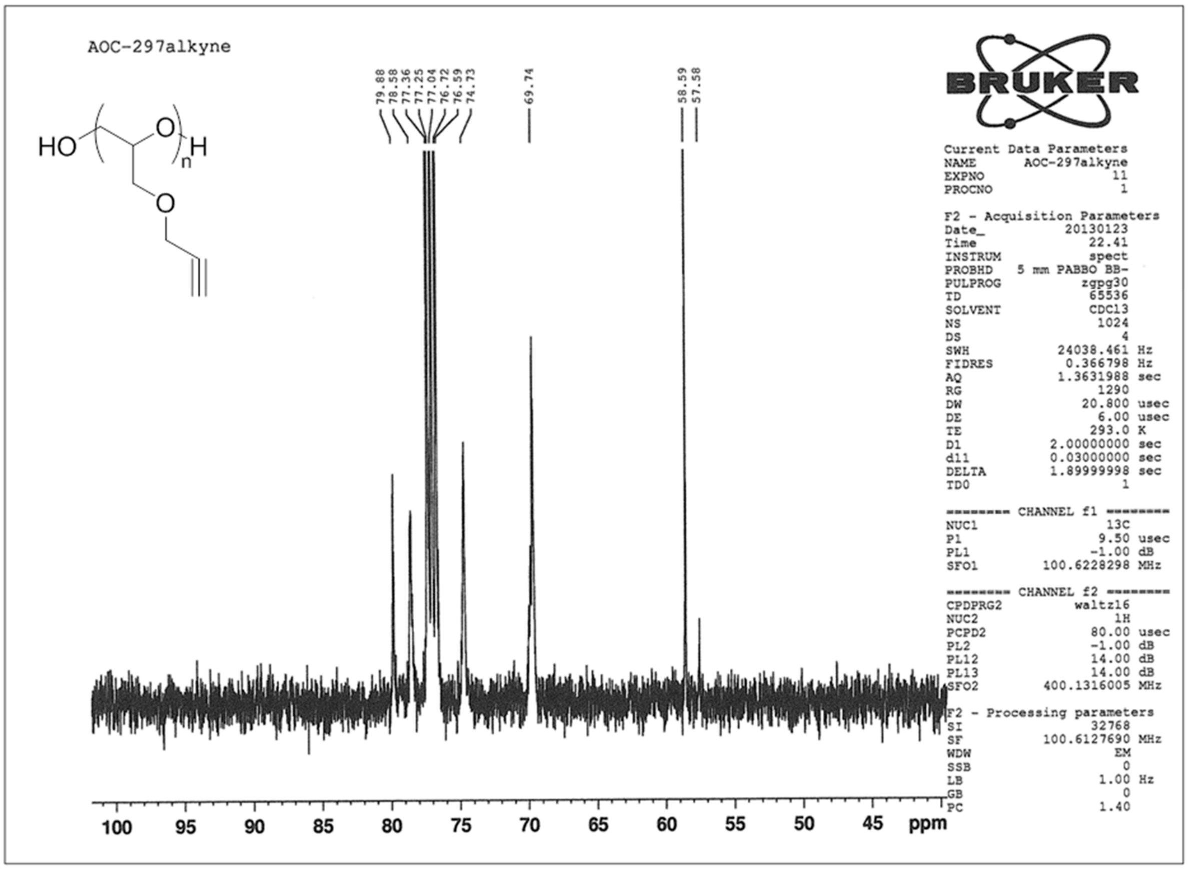

(**b**)

Figure S4. *Cont.*

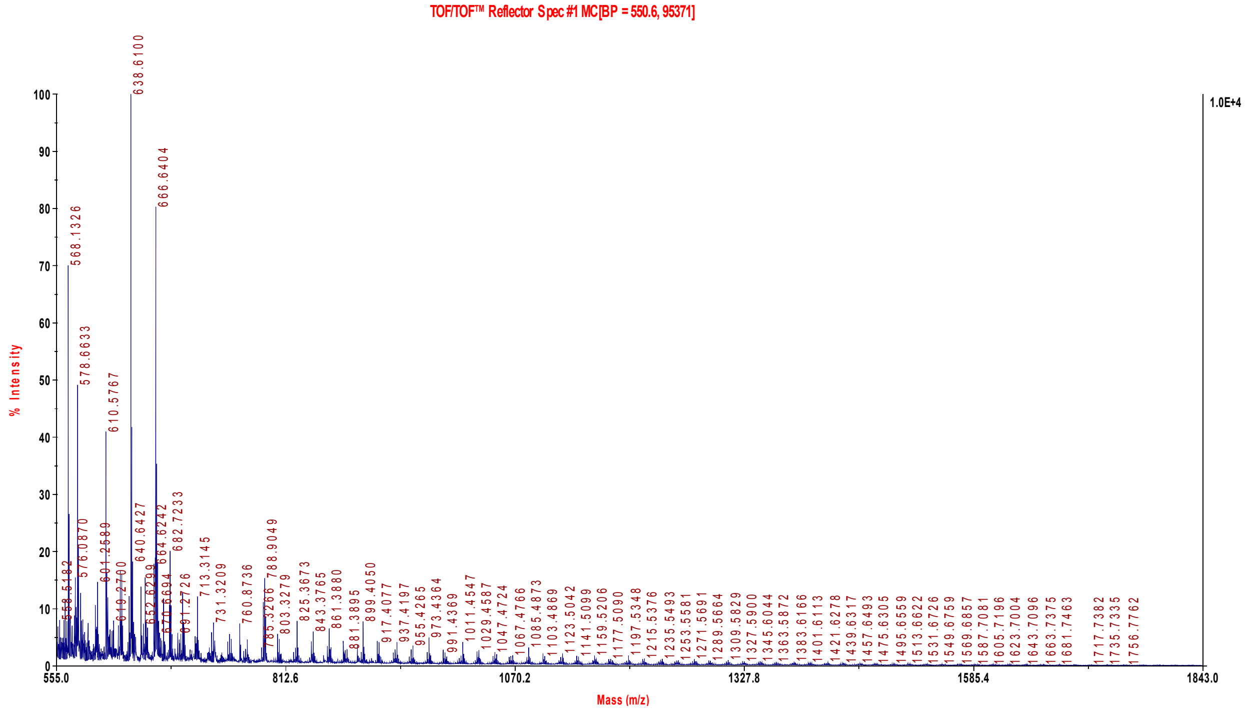

(c)

Figure S5. N3-GGRGD-NH2 (**5**): (**a**) MALDI-TOF MS; (**b**) RP-HPLC.

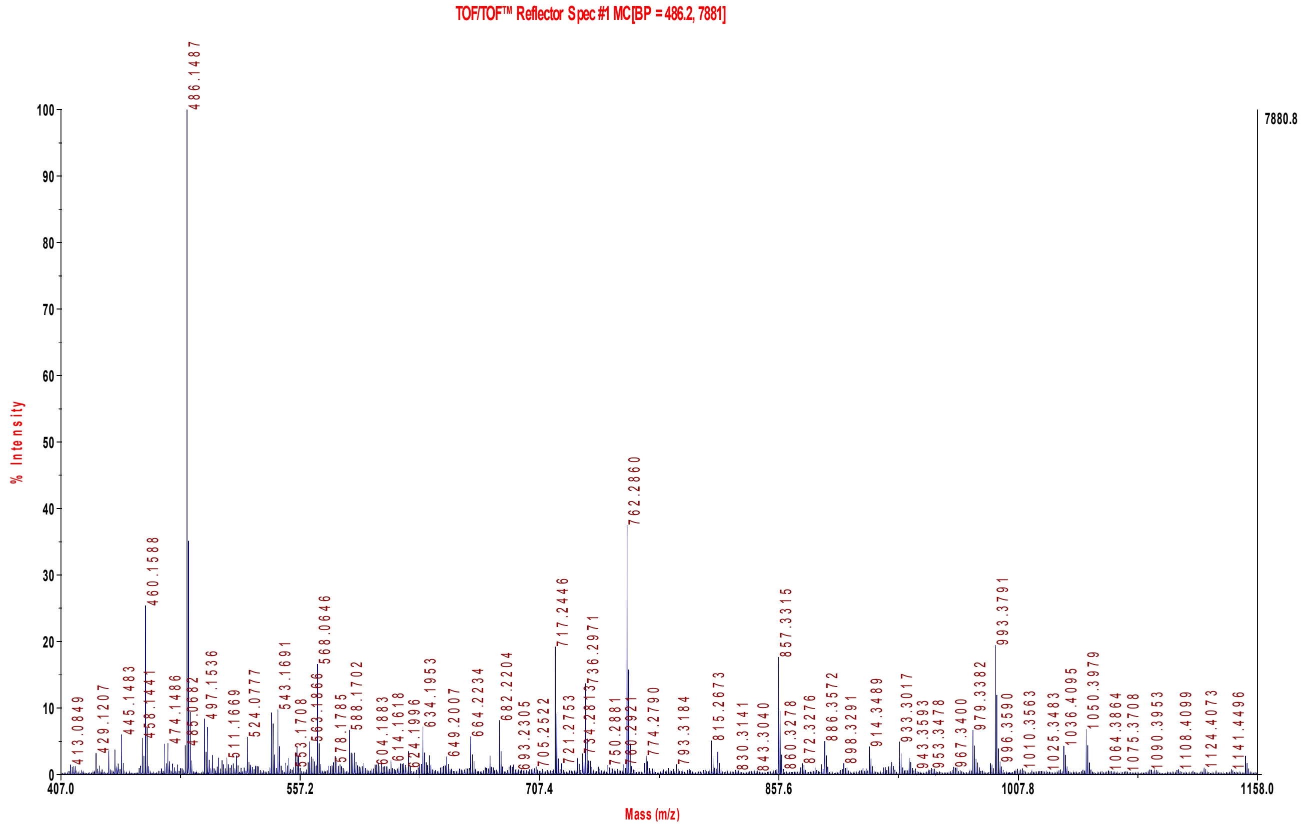

(a)

Figure S5. *Cont.*

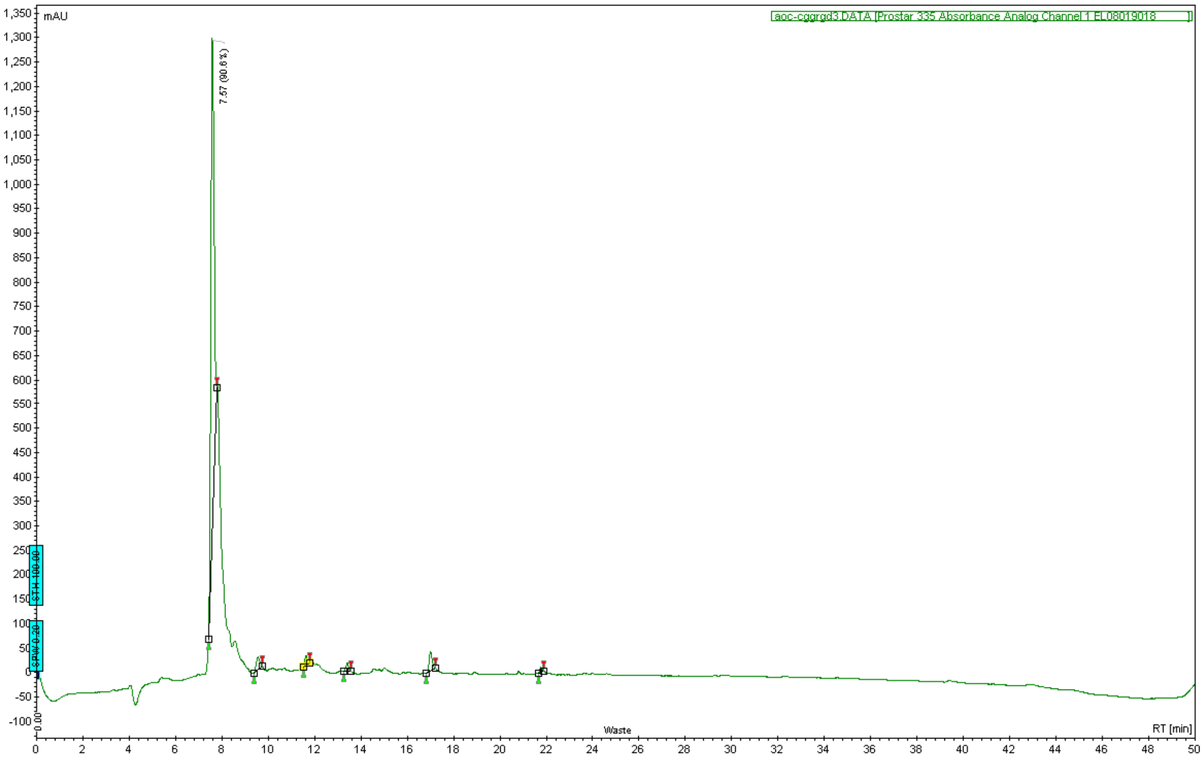

(**b**)

Figure S6. Polyepichlorohydrin (**8**): (**a**) ^{1}H-NMR; (**b**) ^{13}C-NMR; (**c**) MALDI-TOF MS.

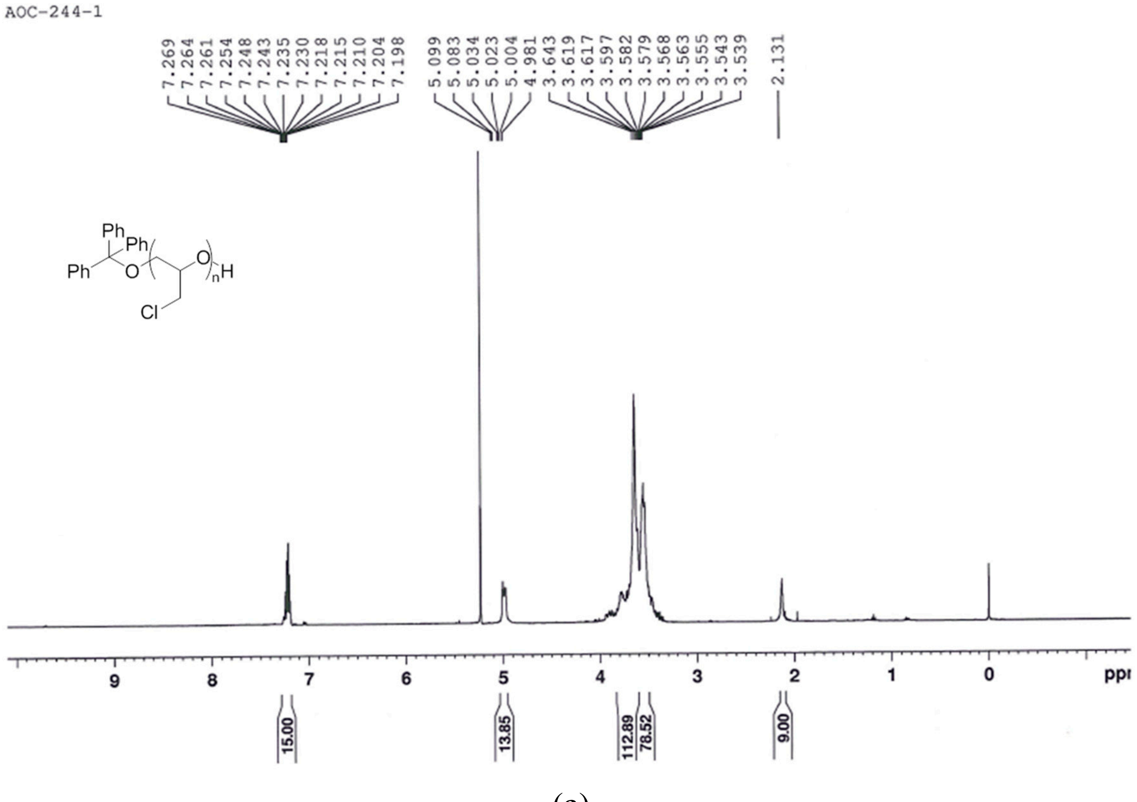

(**a**)

Figure S6. *Cont.*

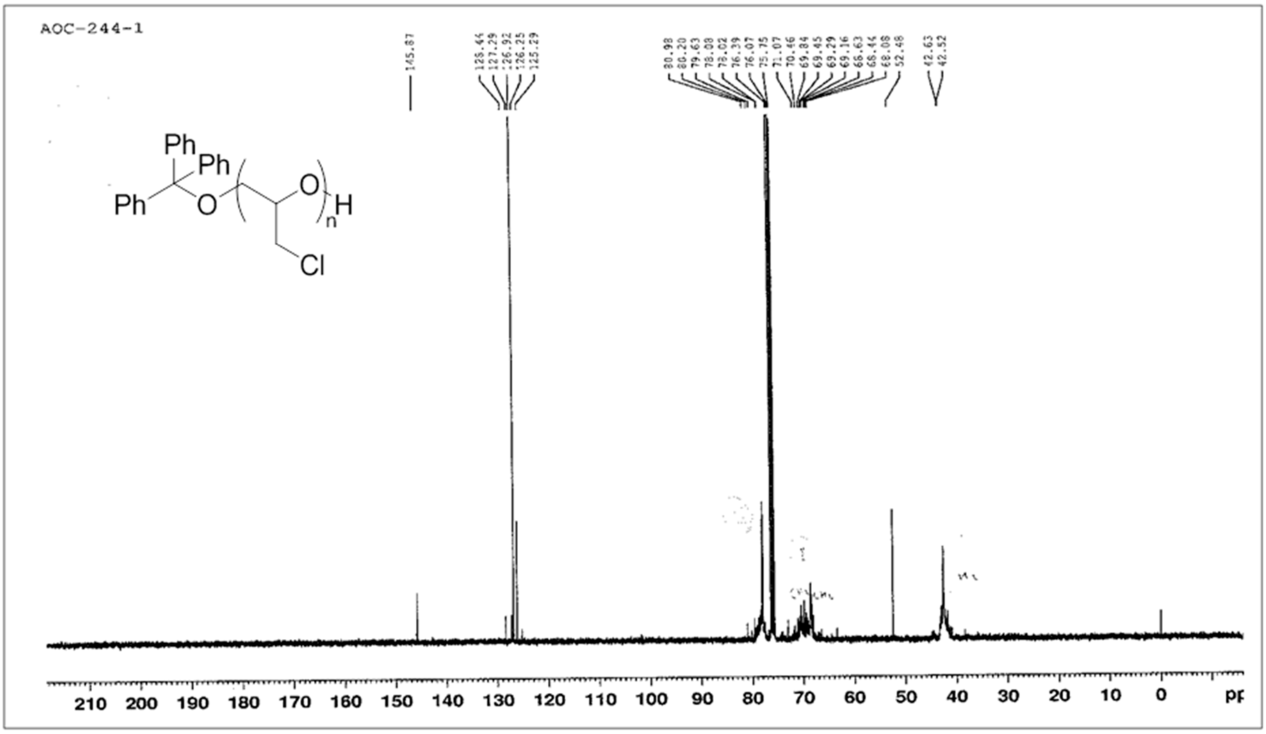

(**b**)

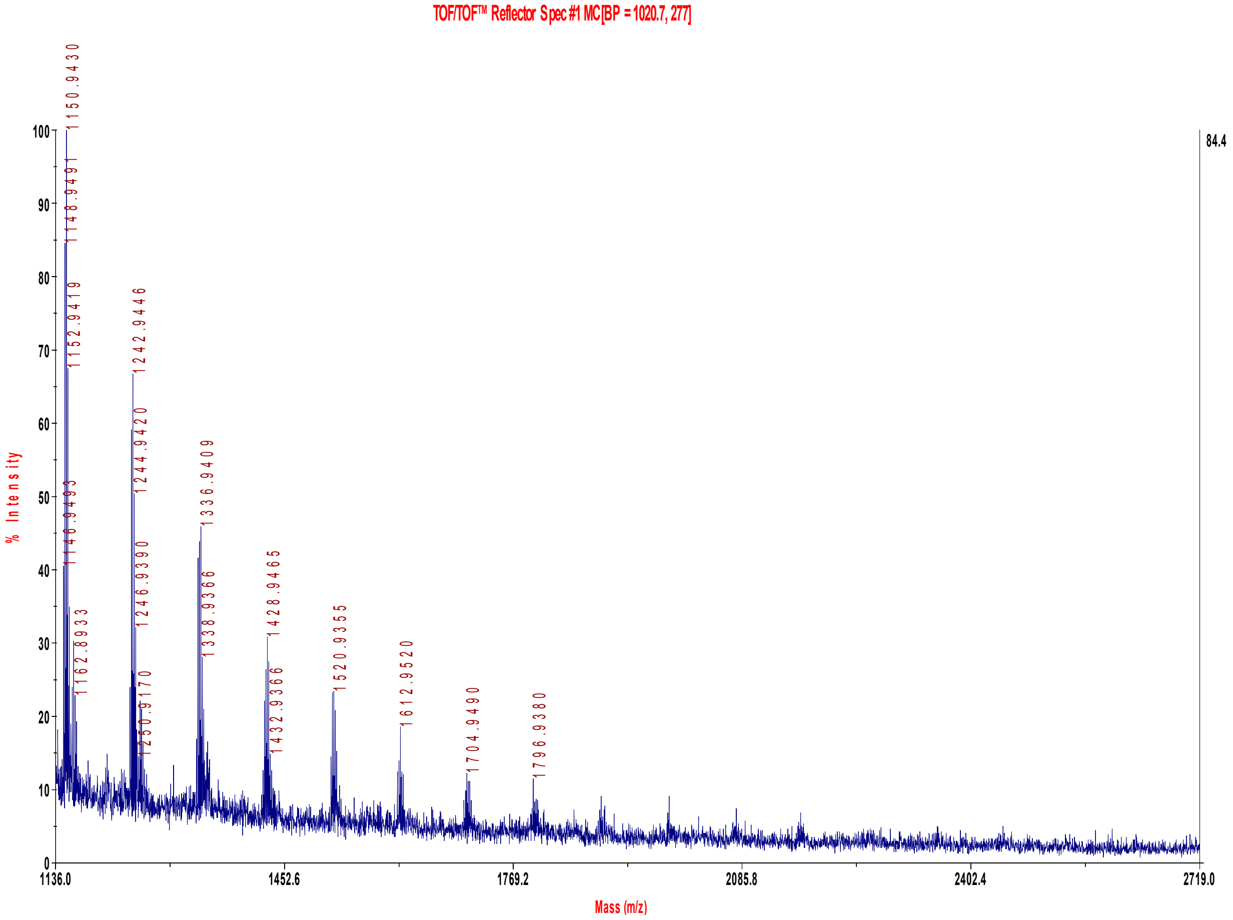

(**c**)

Figure S7. Polyglycidyl azide (**9**). (**a**) ^{1}H-NMR; (**b**) ^{13}C-NMR; (**c**) MALDI-TOF MS.

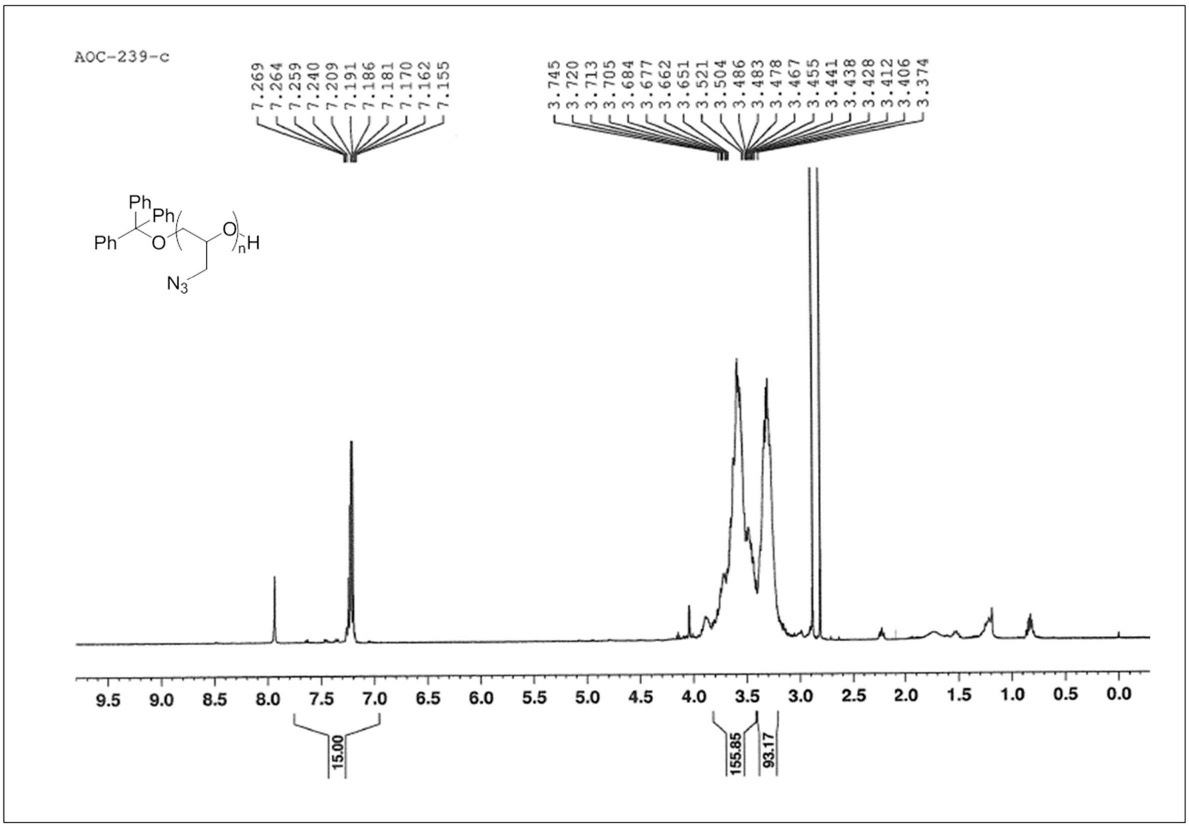

(**a**)

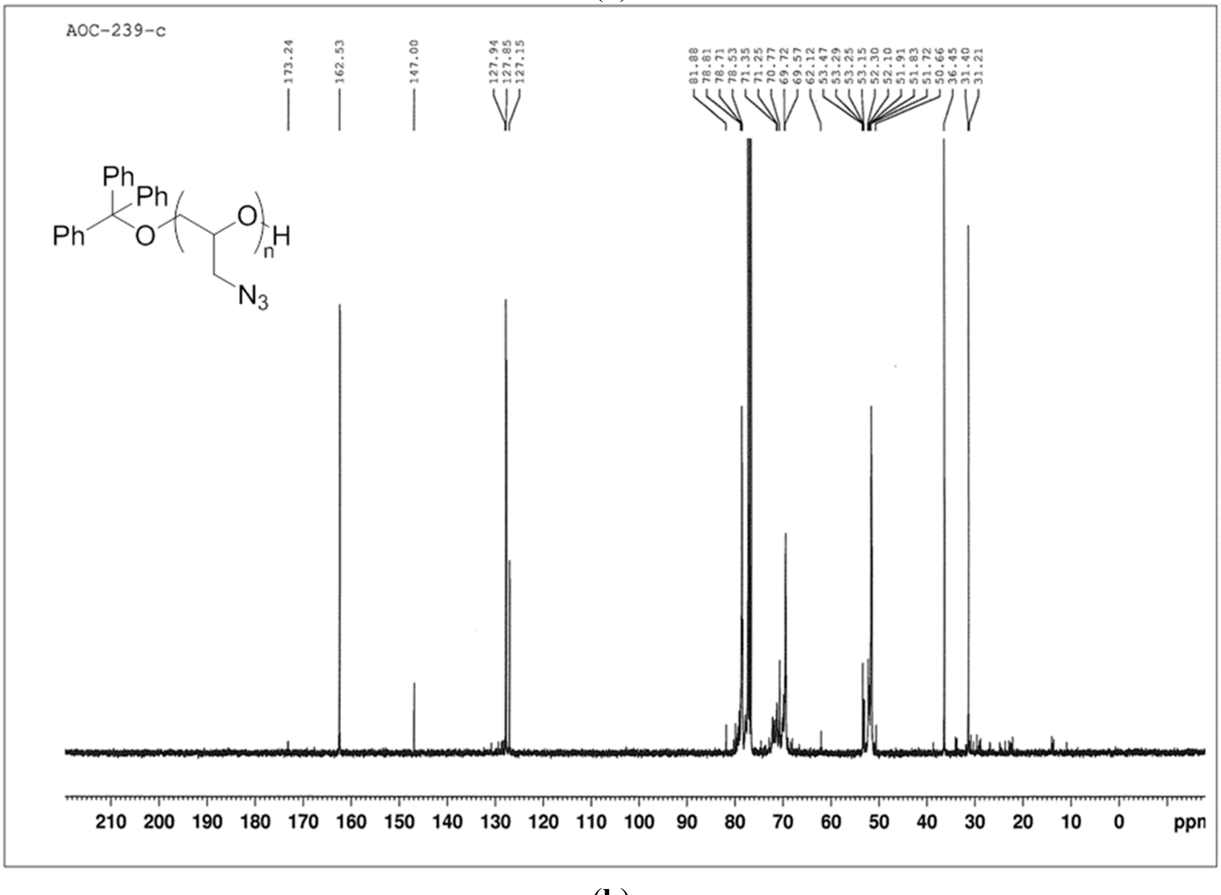

(**b**)

Figure S7. *Cont.*

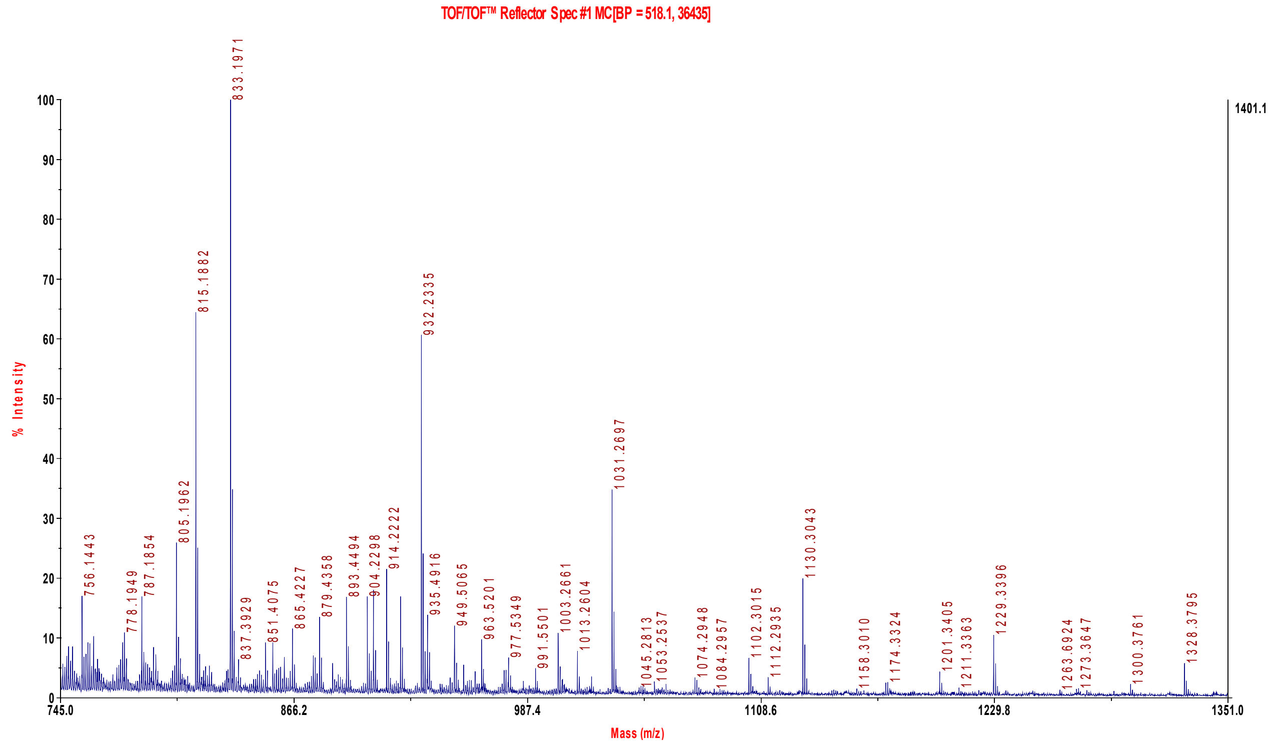

(**c**)

Figure S8. Propiolyl-GWYRGRL-NH_2 (**10**): (**a**) MALDI-TOF MS; (**b**) RP-HPLC.

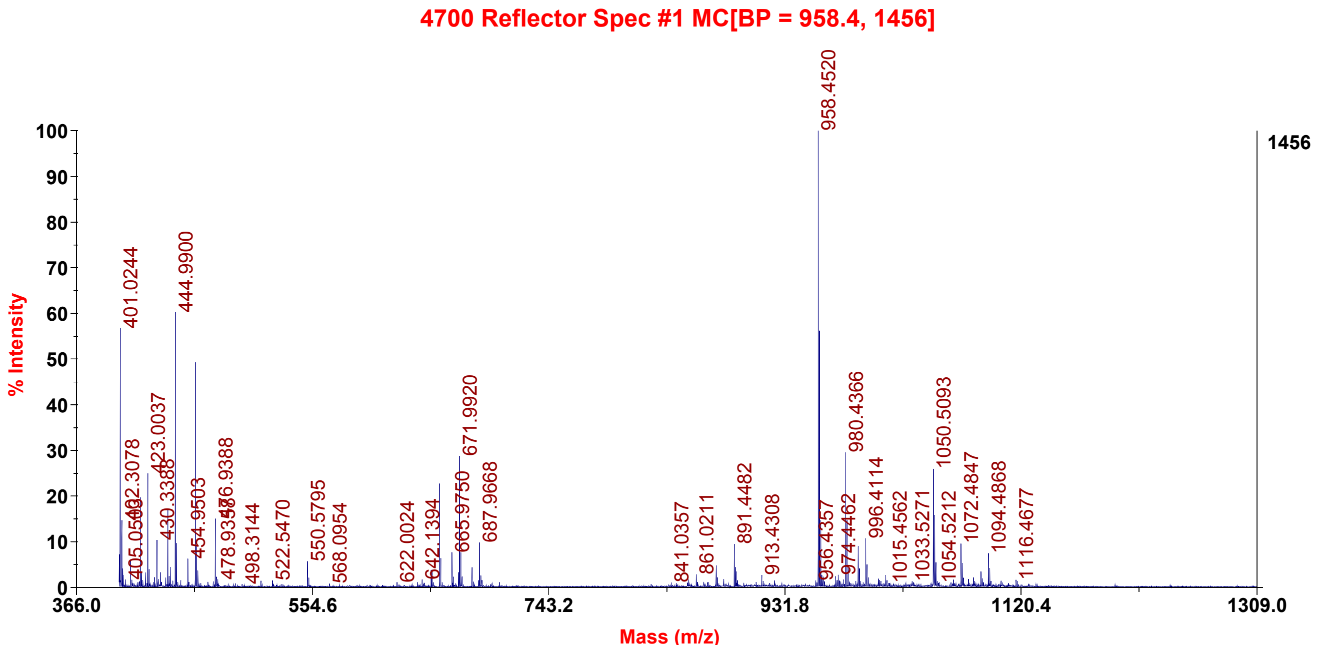

(**a**)

Figure S8. *Cont.*

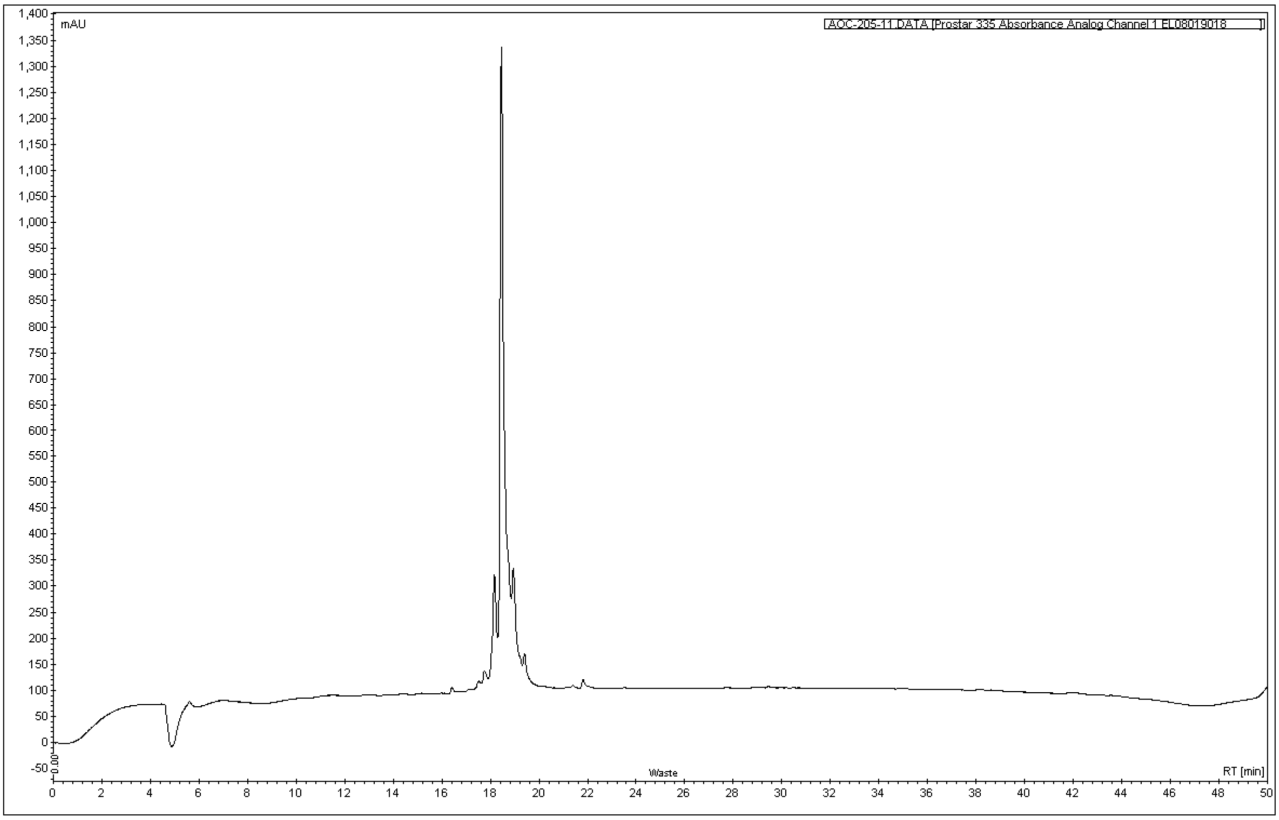

(**b**)

Figure S9. (**11**) Conjugation of (**10**) with polyglycidyl azide (**9**) MALDI-TOF MS.

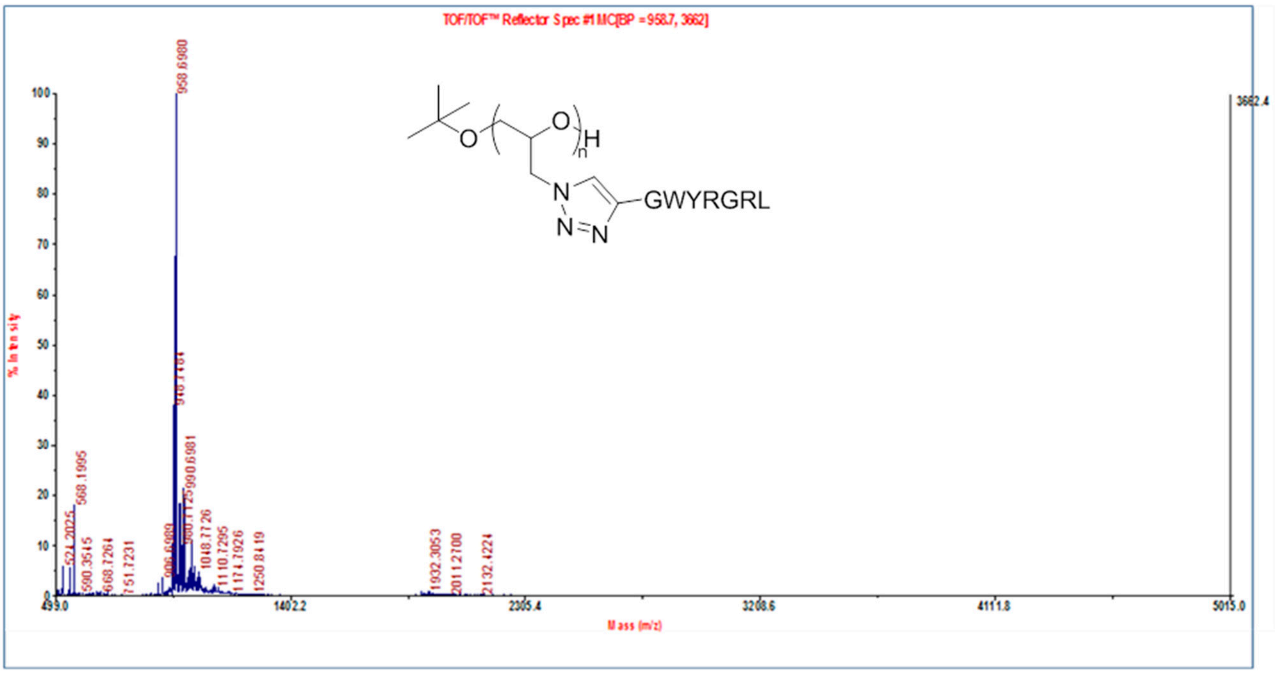

Figure S10. Poly(glyciyl *tert*-butyl carbamate) (**12**): (**a**) ^{1}H-NMR; (**b**) ^{13}C-NMR.

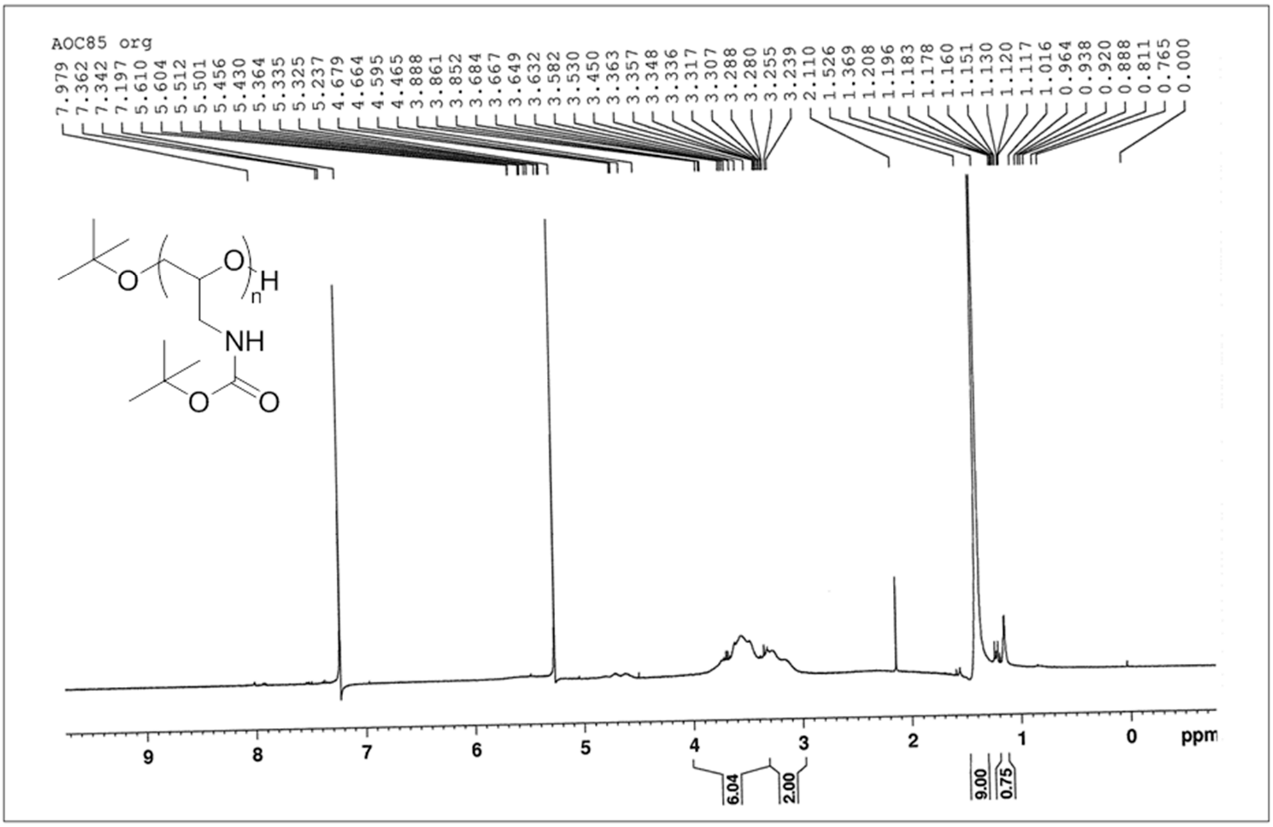

(**a**)

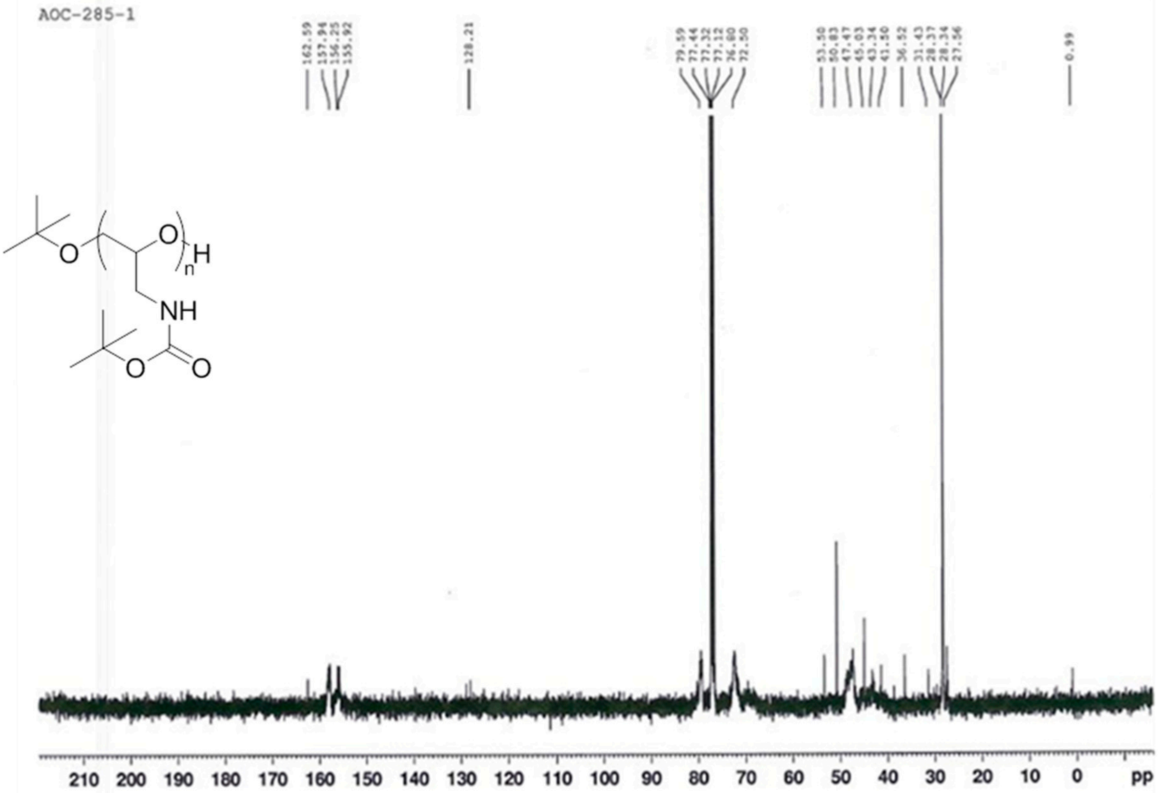

(**b**)

Figure S11. Poly(glycidyl amine) (**13**): (**a**) ^{1}H-NMR; (**b**) ^{13}C-NMR, Dept 135; (**c**) MALDI-TOF MS.

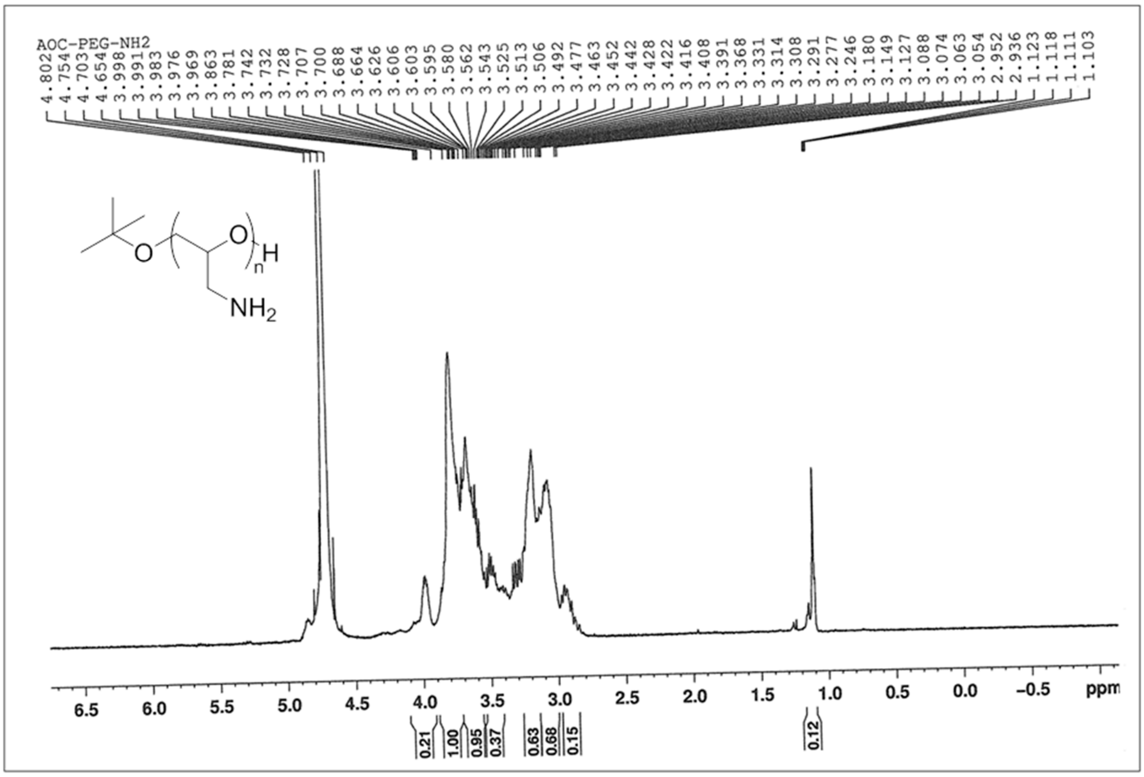

(**a**)

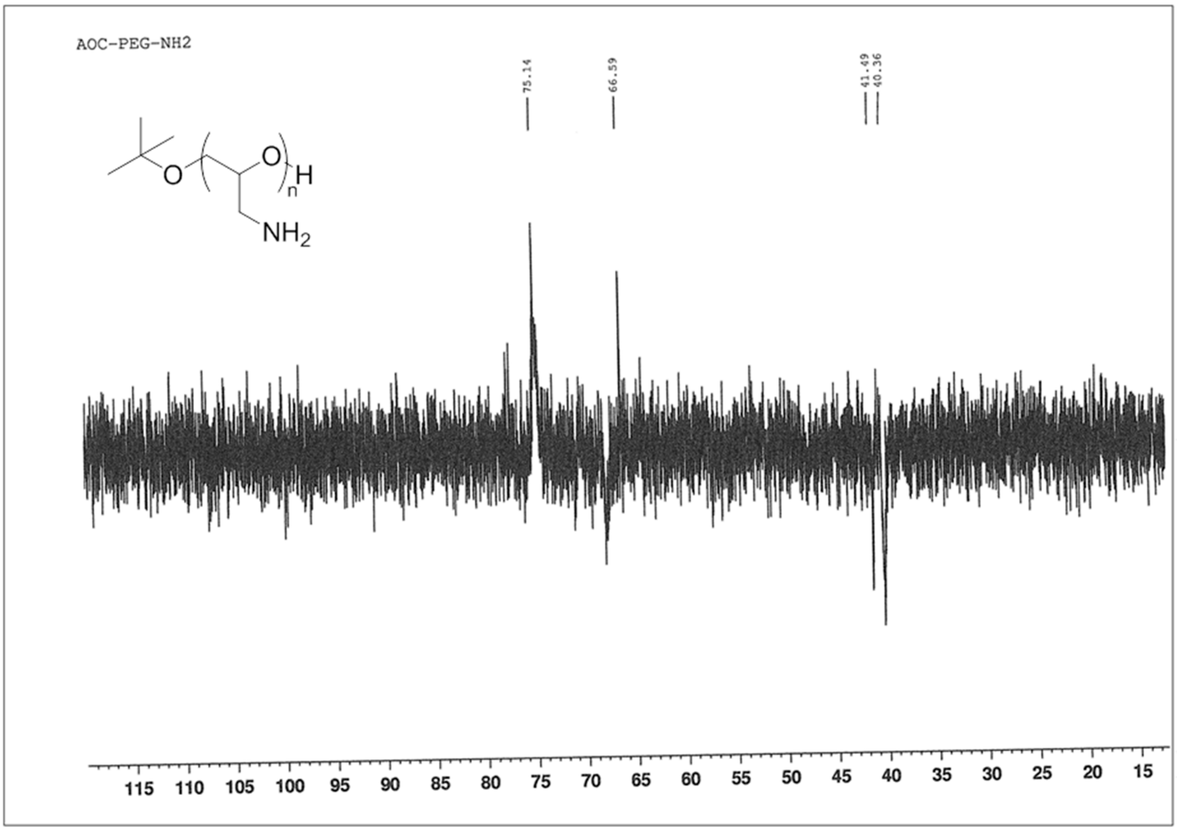

(**b**)

Figure S11. *Cont.*

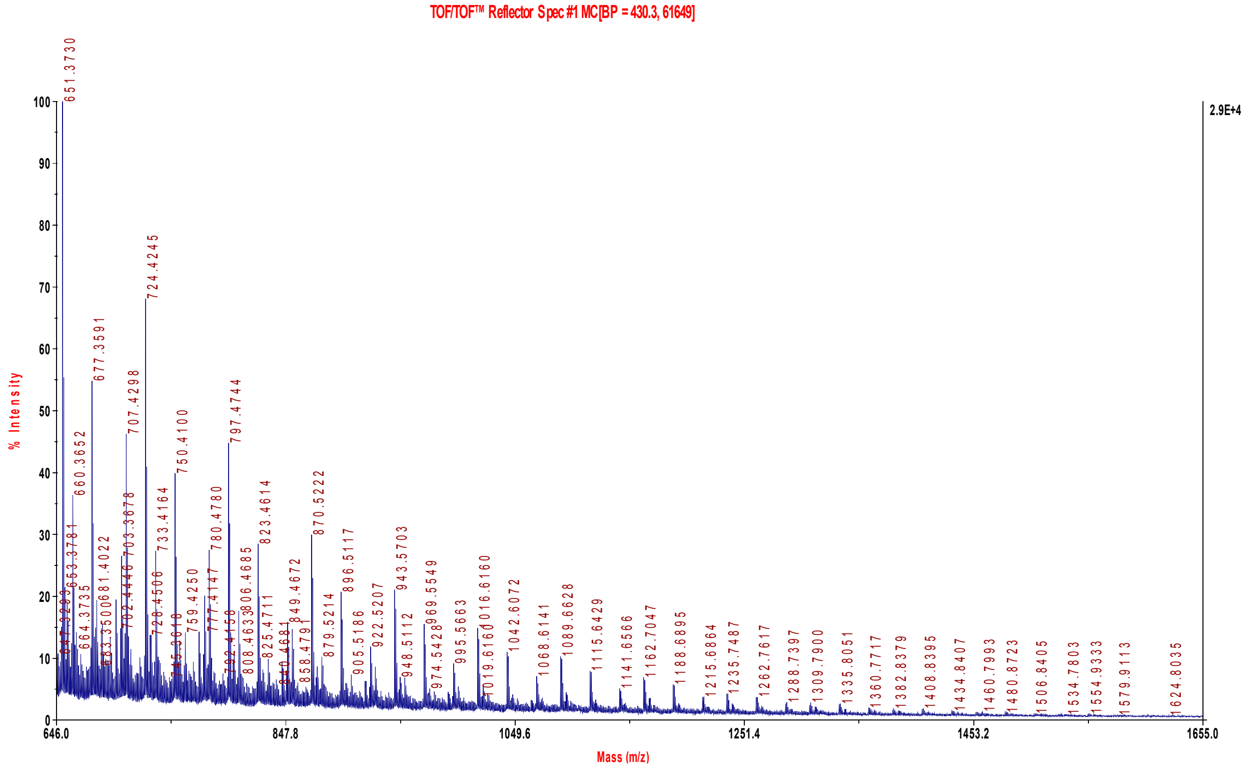

(**c**)

Figure S12. Ac-GWYRGRLE-NH_2 (**14**): (**a**) MALDI-TOF MS; (**b**) RP-HPLC.

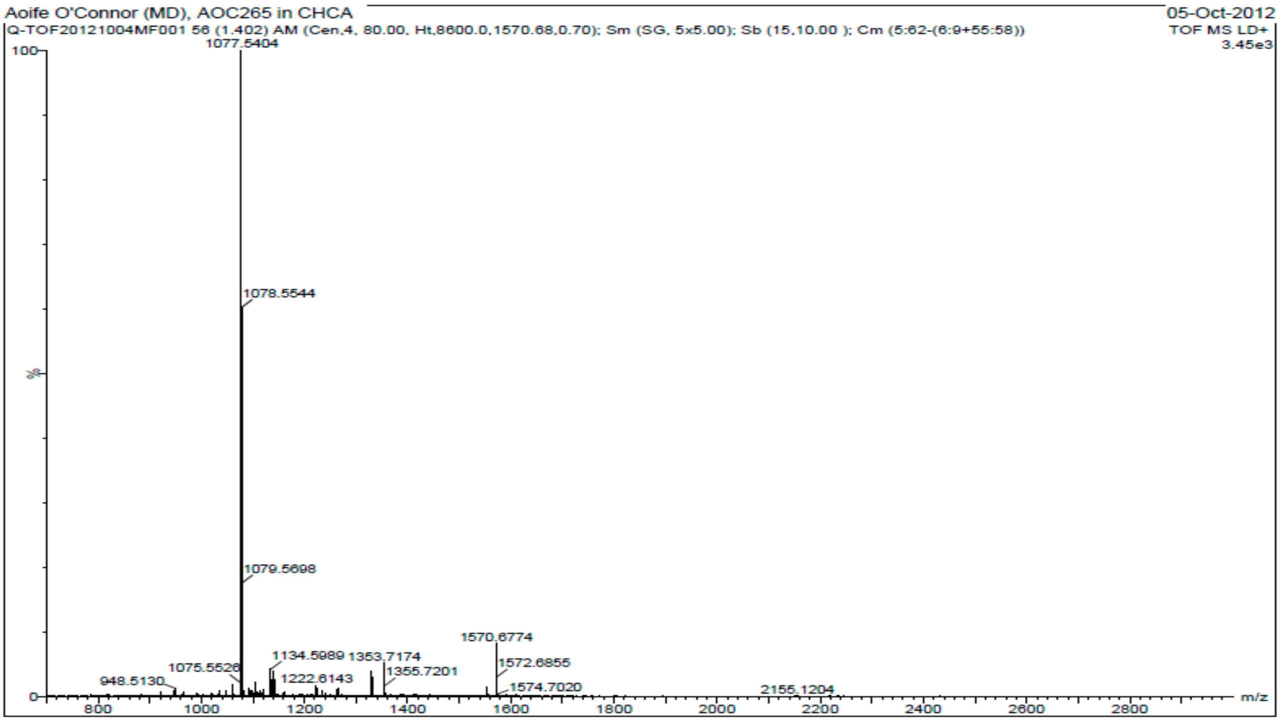

(**a**)

Figure S12. *Cont.*

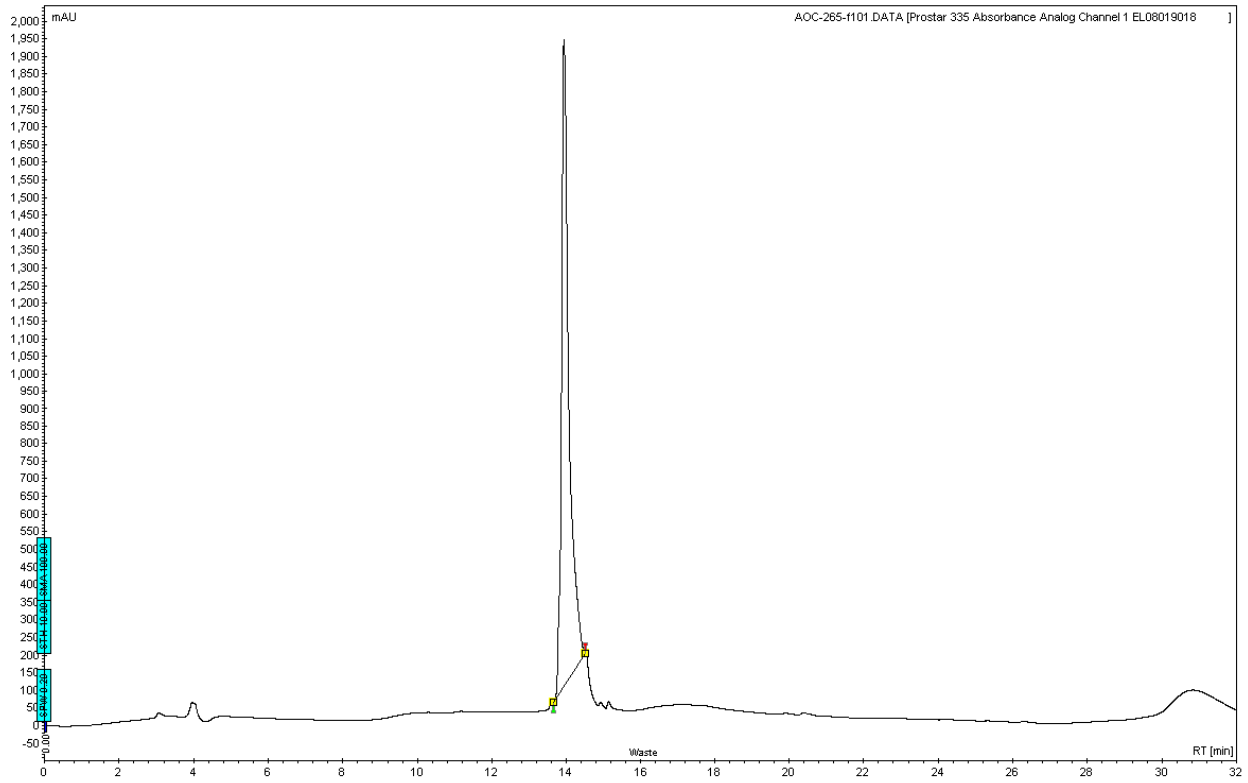

(**b**)

Figure S13. (**15**) Coupling of poly(glycidyl amine) (**13**) to peptide (**14**): (**a**) SEC-HPLC (**15**) (broad peak), compared with (**4**) (narrow peak); (**b**) MALDI-TOF MS of (**15**).

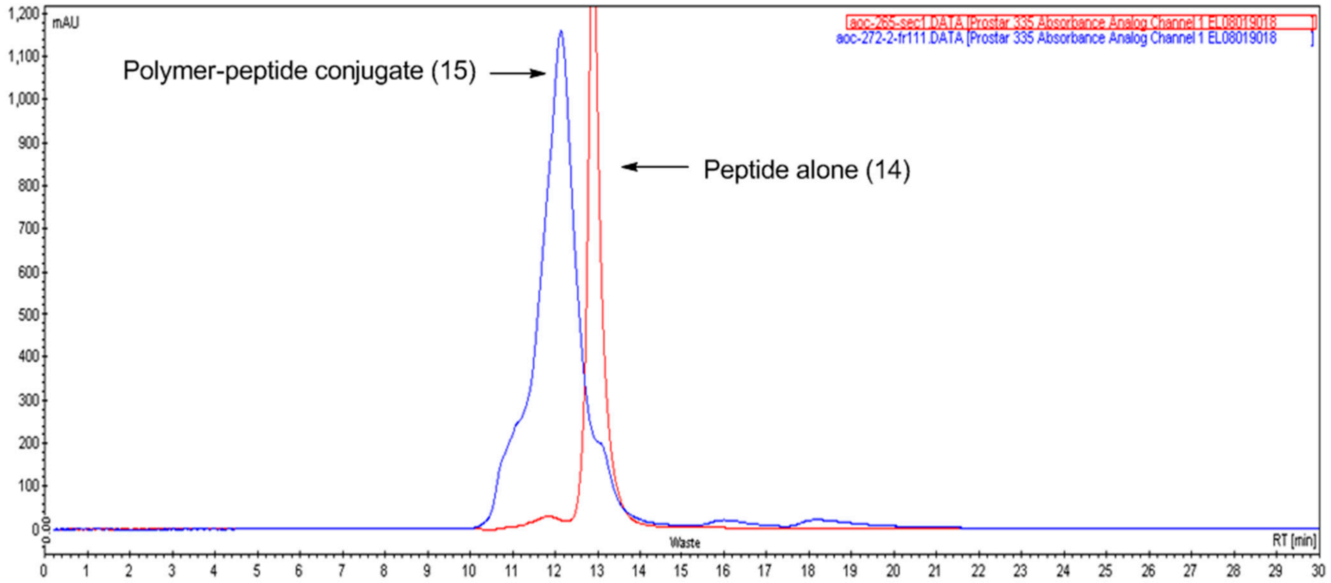

(**a**)

Figure S13. *Cont.*

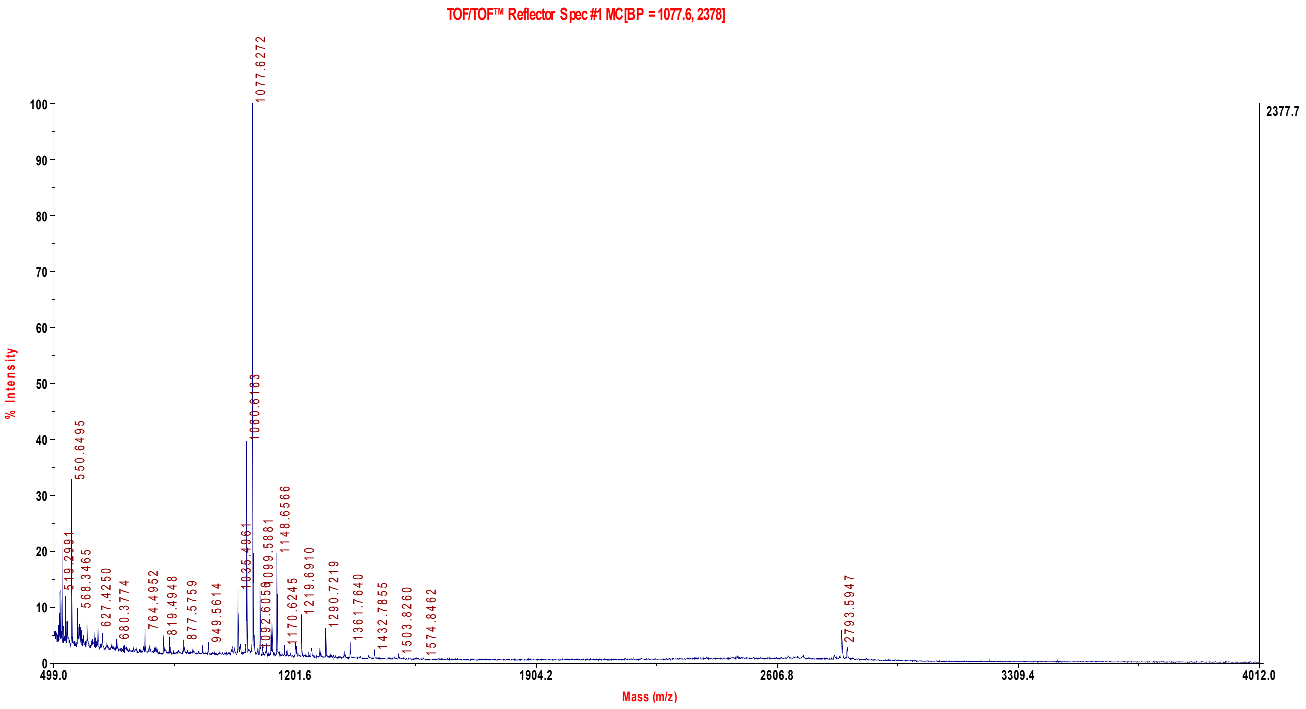

(**b**)

Figure S14. H-CGRGDS-NH2 (**16**): (**a**) MALDI-TOF MS; (**b**) RP-HPLC.

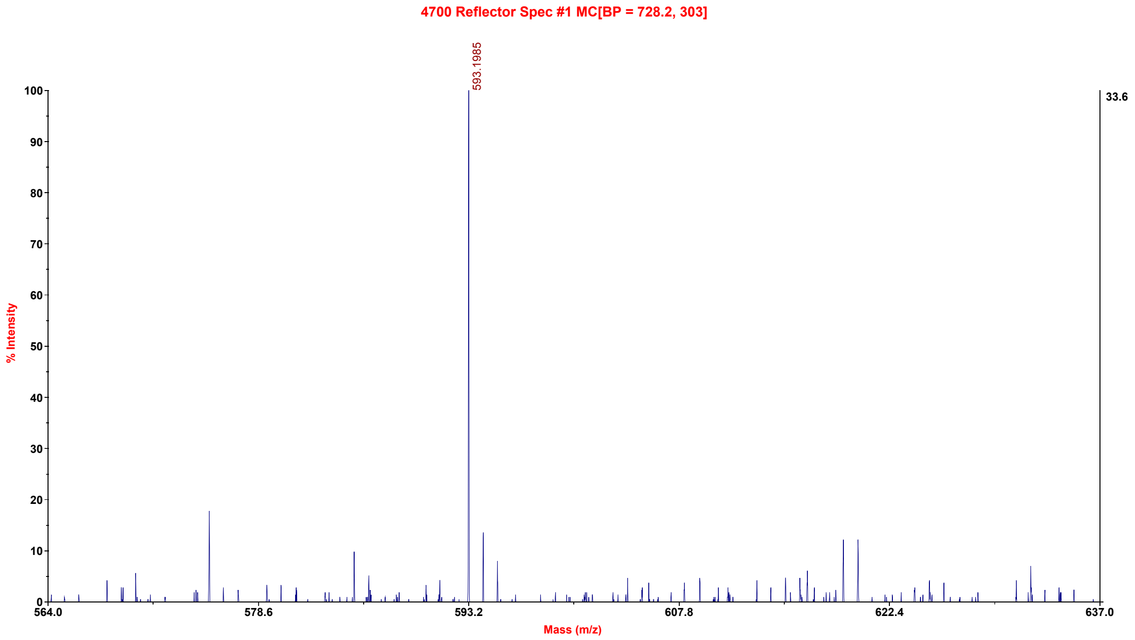

(**a**)

Figure S14. *Cont.*

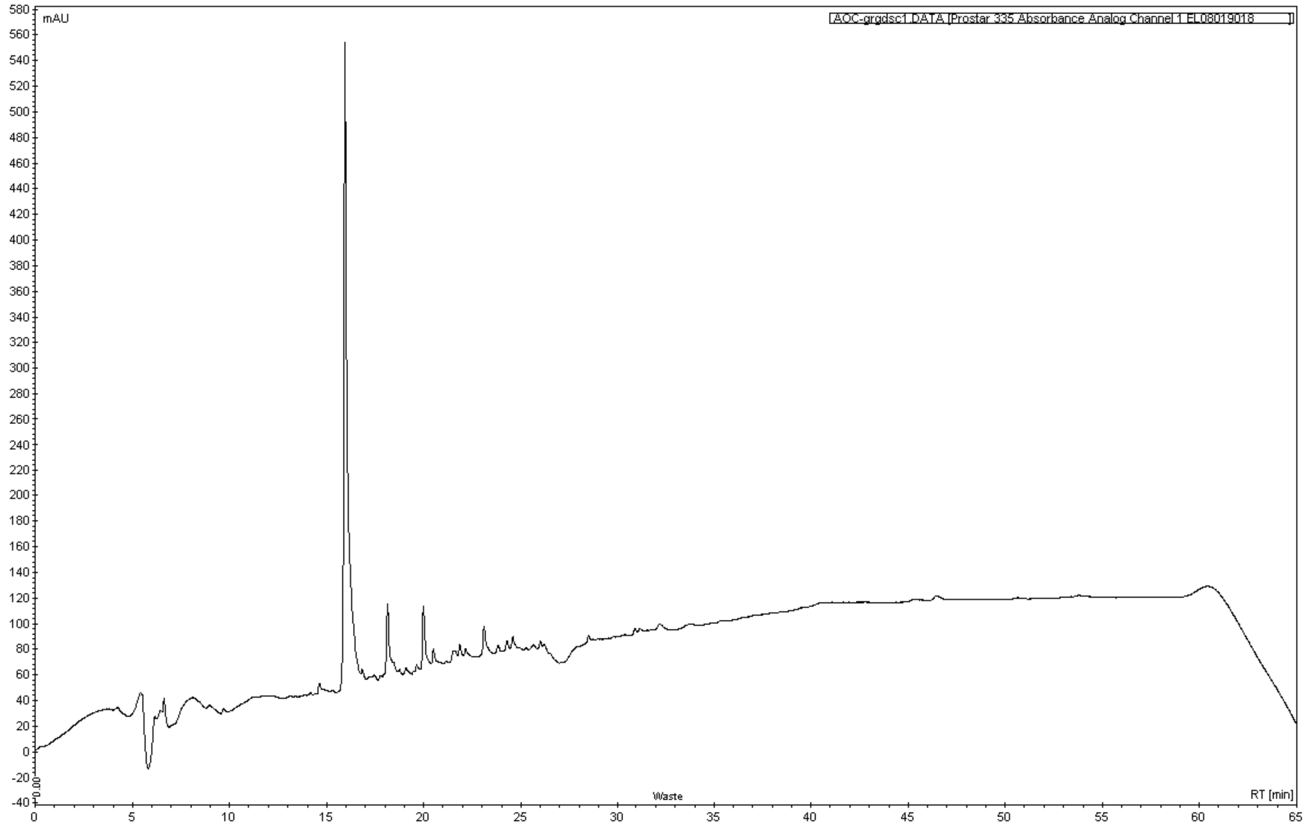

(**b**)

Figure S15. (**17**) Conjugation of H-CGRGDS-NH2 (**16**) and poly(allyl glycidyl ether) (**1**): (**a**) SEC-HPLC of the free peptide (blue trace) and of the product of its conjugation to poly(allyl glycidyl ether) (red trace); (**b**) MALDI-TOF MS analysis of the conjugation product.

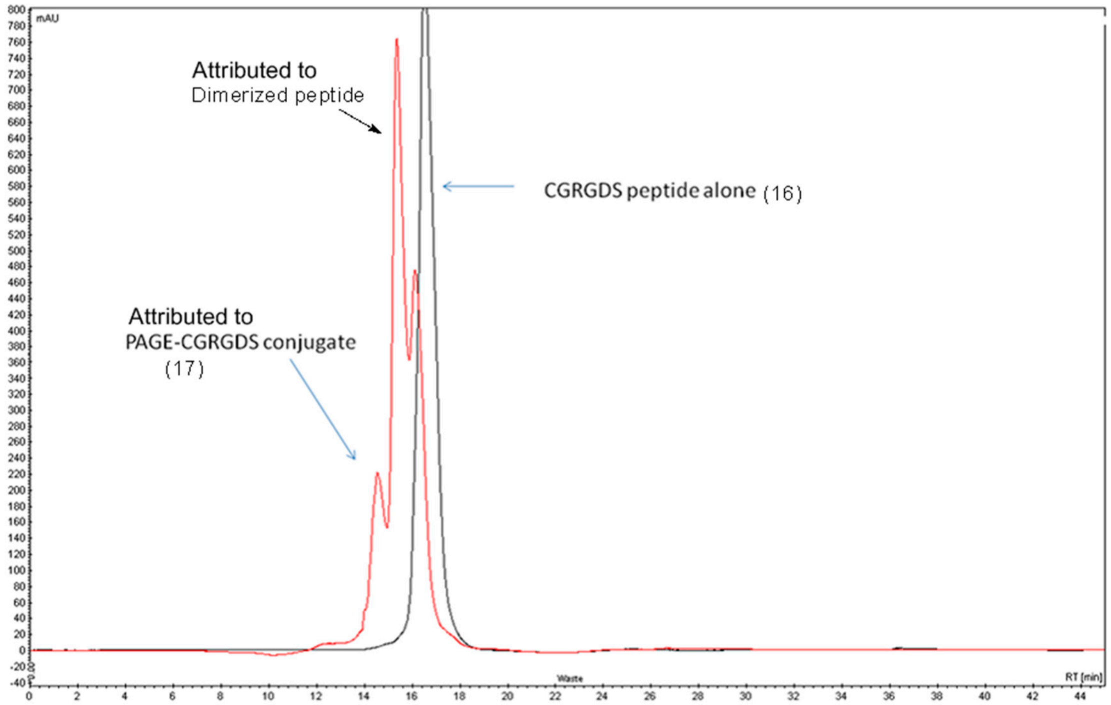

(**a**)

Figure S15. *Cont.*

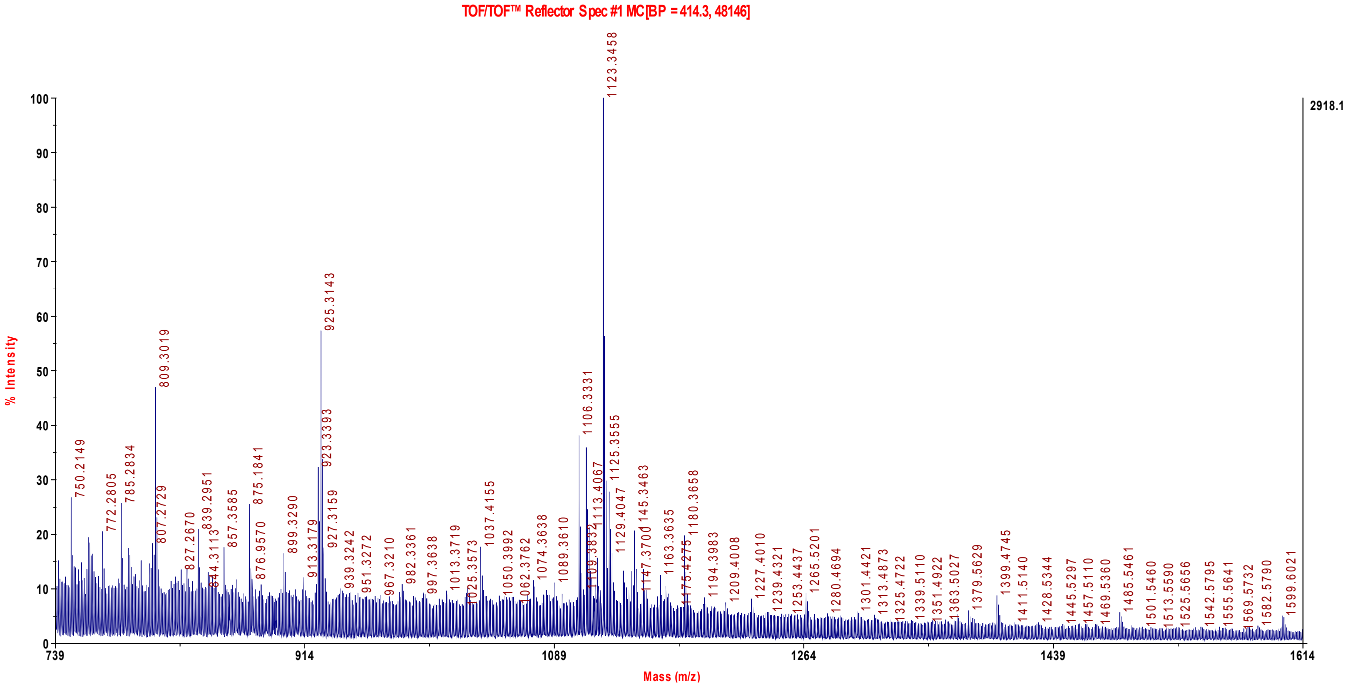

(**b**)
